# The Bioactivity of Thiazolidin-4-Ones: A Short Review of the Most Recent Studies

**DOI:** 10.3390/ijms222111533

**Published:** 2021-10-26

**Authors:** Dominika Mech, Antonina Kurowska, Nazar Trotsko

**Affiliations:** Department of Organic Chemistry, Faculty of Pharmacy, Medical University of Lublin, 20-093 Lublin, Poland; dominika-mech@wp.pl (D.M.); akurowska123@gmail.com (A.K.)

**Keywords:** thiazolidine-4-ones, antioxidant activity, anticancer activity, anti-inflammatory activity, antidiabetic activity, antiparasitic activity, antimicrobial activity, antitubercular activity, multitarget activity, structure-activity relationship

## Abstract

Thiazolidin-4-ones is an important heterocyclic ring system of a pharmacophore and a privileged scaffold in medicinal chemistry. This review is focused on the latest scientific reports regarding biological activities of thiazolidin-4-ones published in 2020 and 2021. The review covers recent information about antioxidant, anticancer, anti-inflammatory, analgesic, anticonvulsant, antidiabetic, antiparasitic, antimicrobial, antitubercular and antiviral properties of thiazolidin-4-ones. Additionally, the influence of different substituents in molecules on their biological activity was discussed in this paper. Thus, this study may help to optimize the structure of thiazolidin-4-one derivatives as more efficient drug agents. Presented information may be used as a practical hint for rational design of new small molecules with biological activity, especially among thiazolidin-4-ones.

## 1. Introduction

Heterocyclic compounds play an important role in many kinds of therapy, where thiazolidin-4-one have been reported to be a potential scaffold to construct new molecules for medicinal chemistry. Thiazolidin-4-one ring is susceptible for modification in Positions 2, 3 and 5. Such modifications capacitate the search for new compounds with desired activity. Literature reported that thiazolidin-4-one is one of the important scaffolds that has therapeutic importance. In case of its modification with other substituents, it shows wide range of biological activities, such as: antidiabetic [1], antioxidant [2], antitubercular [3], antimicrobial [4,5,6,7], anticonvulsant [8] anticancer [9,10,11], antiprotozoal [12,13] and anti-inflammatory activities [14,15]. Additionally, thiazolidine-2,4-diones are well-known group of antidiabetic drugs (Pioglitazone, Rosiglitazone etc.) that reveal affinity to PPARγ [16,17].

The appearance of new information about the activity of thiazolidin-4-ones requires regular systematization and analysis. Therefore, in this review, we present the range of biological applications for thiazolidin-4-one derivatives published in 2020 and 2021. In addition, we outline the effect of substituents on their biological activity.

## 2. Biological Activities of Thiazolidin-4-Ones

### 2.1. Antioxidant Activity

Free radicals are important factors causing many pathological processes in the human body including the development of diseases of civilization. They can damage cell membranes, proteins, enzymes and DNA, which increase the risk of many conditions such as Parkinson’s disease, Alzheimer’s disease, angiocardiopathies, asthma, diabetes, atherosclerosis, eye degenerative diseases, chronic inflammation, neurodegenerative diseases and some types of cancer [18]. Reactive forms of oxygen and nitrogen are responsible for the oxidative deterioration of the quality of food products. Hence, nowadays there is a huge interest in compounds with antioxidant activity. The following studies demonstrate the effects of thiazolidine derivatives in this direction as well, taking into account how various substituents may condition and modulate antioxidant activity.

**Figure 1:**
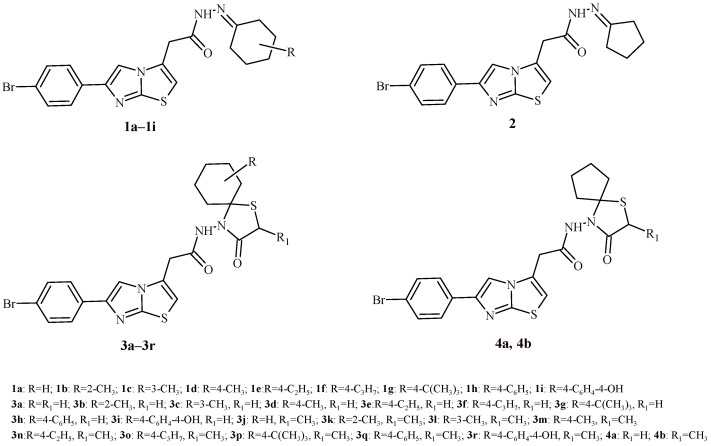
Imidazo[2,1-*b*]thiazole-thiazolidin-4-one hybrids (**3a**–**3r**, **4a** and **4b**) and their precursors (**1a**–**1i** and **2**) with antioxidant activity.

In 2020, a group of researchers published a paper which showed how certain substituents to the thiazolidine heterocyclic ring may be responsible for its adaptation to the human peroxiredoxin 5 enzymes [19]. This enzyme plays an important role in the process of fighting free radicals and protecting against oxidative stress. The antioxidant activity of Compounds **1a**–**1i**, **2**, **3a**–**3r**, **4a** and **4b** on lipid peroxidation were evaluated by TBARS assay (Figure 1). The studies showed that the substitution of cyclohexyl moiety at Position 4 by the 4-hydroxyphenyl substituent (Compounds **3i** and **3r**) significantly increased the antioxidant activity in the structures. The Compounds **3i** and **3r** showed the best inhibitory activity of lipid peroxidation with EC_50_ 0.565 ± 0.051 and 0.708 ± 0.074 mM, respectively. It has been shown that the lack of substitution at the R_1_ position increases the activity to a maximum (Compound **3i**). The precursor of thiazolidine-4-one derivatives (**3a**–**3r**) Compounds **1a**–**1i** showed antioxidant activity with EC_50_ in the range of 1.128–2.489 mM. Compounds containing a cyclopentyl moiety, Compounds **2** (EC_50_ = 4.156 ± 0.178 mM), **4a** (EC_50_ = 3.30 ± 0.271 mM) and **4b** (EC_50_ = 9.388 ± 0.911 mM), showed the lowest inhibitory capacity against lipid peroxidation among all tested derivatives.

In addition, it was concluded from the modelling studies that the hydrogen bond interactions between the ligands and amino acids Cys47, Arg127, Thr44, Thr147, Gly46, Asp113 of the human peroxiredoxin 5 (PDB ID: 1HD2) showed significant antioxidant activity. Additionally, the overlapping of π-π orbitals between the ligands and the receptor-building amino acid Phe120 may play a role in the process of action.

Another study contemplating on antioxidant activity of thiazolidine-2,4-dione (TZD) derivatives was published in 2020 by Kumar et al. [20]. The antioxidant activity of the 5-(-4-((substituted aryl/alkyl)methyl)benzylidene)thiazolidine-2,4-dione was assessed by applying DPPH radical scavenging method. Ascorbic acid was used as standard drug.

**Figure 2:**
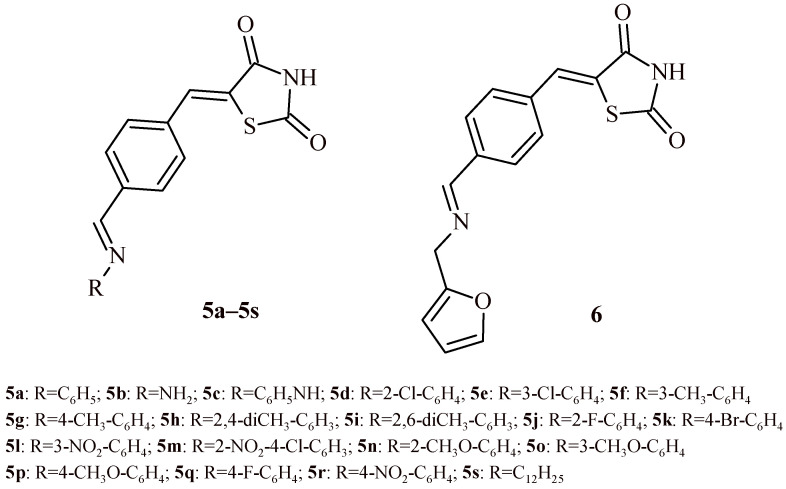
The 5-Arylidenethiazolidine-2,4-dione derivatives (**5a**–**5s**) and **6** with antioxidant and antimicrobial activity.

The antioxidant test showed that all twenty synthesized compounds (**5a**–**5s** and **6**)—were more active than the reference drug—ascorbic acid (Figure 2). Their IC_50_ was in the range of 9.18–32.43 µg/mL. The most active derivative among all tested compounds was Derivative **6**. The Compound **6** with (furan-2-ylmethyl)imino substituent showed prominent antioxidant activity results (IC_50_ = 9.18 µg/mL) compared to the reference drug (IC_50_ = 40 µg/mL). Additionally, Compounds **5c**, **5d**, **5i**, **5r** and **5s** were 2-fold more active than ascorbic acid with IC_50_ values in the range of 12.67–18.02 µg/mL. Whereas Compound **5m** with two electron-withdrawing groups (NO_2_ and Cl) in the benzene ring exhibited the lowest antioxidant activity with IC_50_ = 32.43 µg/mL.

**Figure 3:**
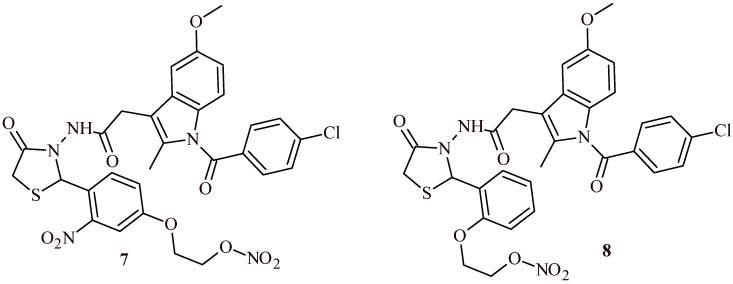
Thiazolidin-4-one-indometacin hybrids with antioxidant properties.

Sava et al. conducted synthesis of thiazolidin-4-one-indometacin hybrids and evaluated their antioxidant activity with use of DPPH radical scavenging method [21]. The most active derivatives from whole series were Compounds **7** and **8** with IC_50_ values 0.54 ± 0.01 and 1.82 ± 0.05 mM, respectively (Figure 3). Moreover, vitamin C equivalent antioxidant capacity (CEAC) value was determined. The CEAC value show how many times tested compounds are more active than vitamin C. All tested compounds were less active than vitamin C used as positive control. The most potent derivative was Compound **7** with the CEAC value 0.137 ± 0.01 vs 1 for vitamin C. Furthermore, Compound **7** revealed around 100-fold more activity than indometacin (CEAC value 0.0014 ± 0.0002).

**Figure 4:**
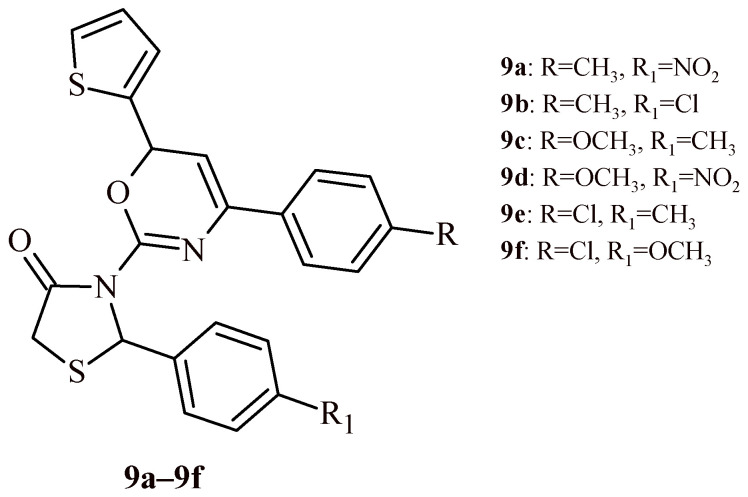
The oxazinyl-thiazolidin-4-ones (**9a**–**9f**) with antioxidant activity.

The series of oxazinyl-thiazolidin-4-ones (**9a**–**9f**) were tested for their antioxidant activity in DPPH and nitric oxide (NO) radical scavenging activity assays (Figure 4) [22]. The Compound **9a** showed highest antioxidant activity with IC_50_ values of 6.62 µg/mL (DPPH assay) and 6.79 µg/mL (NO assay). Compound **9c** was also worthy of attention because it exhibited antioxidant activity at concentration 9.33 and 6.05 µg/mL, correspondingly for DPPH and NO tests. Other derivatives showed less antioxidant activity than standard ascorbic acid (IC_50_ values of 22.88 µg/mL for DPPH assay and 12.61 µg/mL for NO test).

**Figure 5:**
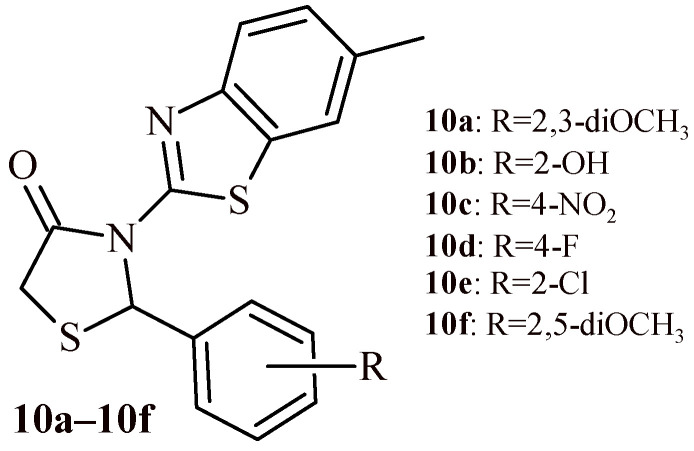
The thiazolidin-4-one-benzothiazole hybrids with antioxidant activity.

Shetty et al. synthesized thiazolidin-4-one-benzothiazole hybrids (**10a**–**10f**) and conducted their evaluation for antioxidant activity (Figure 5) [23]. For evaluation of antioxidant activity three different models (DPPH, nitric oxide and superoxide anion radical scavenging assays) were used. The most effective compound was Derivative **10d** with IC_50_ values of 30.9 µg/mL (DPPH), 33.82 µg/mL (NO) and 31.88 µg/mL (Superoxide anion). However, these concentrations were higher than those for ascorbic acid used as standard (IC_50_ values in the range of 24.99–25.73 µg/mL).

**Figure 6:**
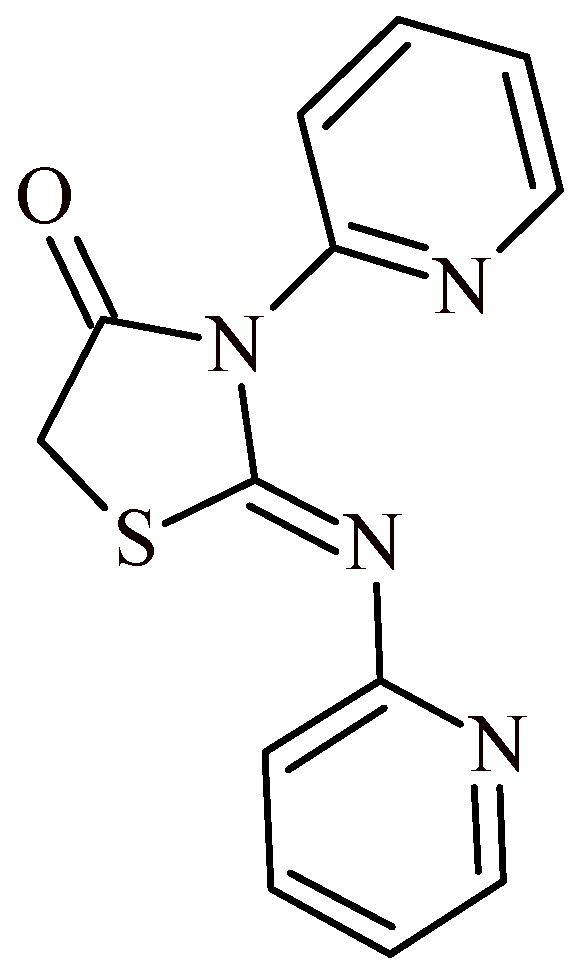
The structure of 3-(pyridin-2-yl)-2-(pyridine-2-ylimino)thiazolidin-4-one (PPIT).

Carraro Junior et al. conducted evaluation of 3-(pyridin-2-yl)-2-(pyridine-2-ylimino)thiazolidin-4-one (PPIT) for their antioxidant activity and inhibition potential of monoamine oxidase (MAO) [24]. The studies demonstrated that PPIT (Figure 6) has antioxidant and reducing activity according to the ABTS, DPPH, FRAP and PC assays. Additionally, PPIT selectively inhibited the activity of cerebral MAO-B isoform in concentration equal to or greater than 200 µM. The compound PPIT inhibited MAO-A isoform in none of the tested concentrations.

**Figure 7:**
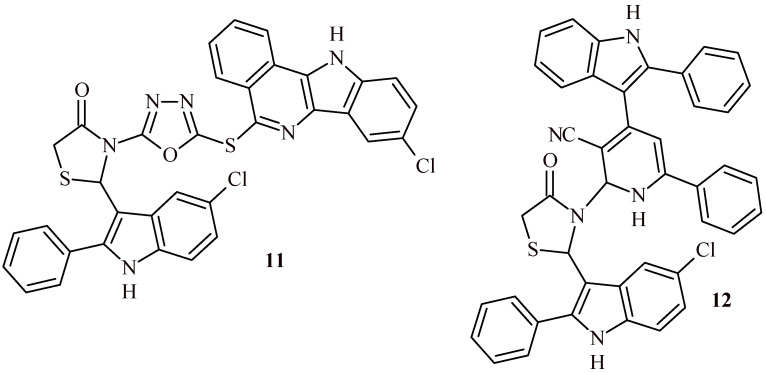
The structures of Compounds **11** and **12**.

Verma et al. reported that Compound **11** (Figure 7) demonstrated excellent radical scavenging activity (78.83% of inhibition at concentration 100 µg/mL). The evaluation was carried out by the DPPH test [25]. Compound **12** with indolyl-pyridine moiety in structure showed more potent radical scavenging activity than **11** (80.89%, 86.02%, 89.86% and 92.05 % at concentration 25, 50, 75 and 100 µg/mL, respectively) [26].

### 2.2. Anticancer Activity

Malignant neoplasms are diseases that occur commonly in our population and have a relatively high mortality rate. It is the second leading cause of death in developed countries, after cardiovascular disease. For many years, both the morbidity and mortality rate from malignant neoplastic diseases have been increasing. Only in recent years this tendency is slowed down [27]. The background to this state of affairs is the change in the demographic structure of societies over the years as well as the exposure to carcinogens, not all recognized to this day. In Poland, there are 155,000 cases and 93,000 deaths annually [28].

Vascular endothelial growth factor (VEGF) is a well-characterized pro-angiogenic factor, necessary for the formation of new blood vessels during both embryonic development and pathological conditions. Research conducted recently has indicated a new role of VEGF as a neurotrophic factor [29].

**Figure 8:**
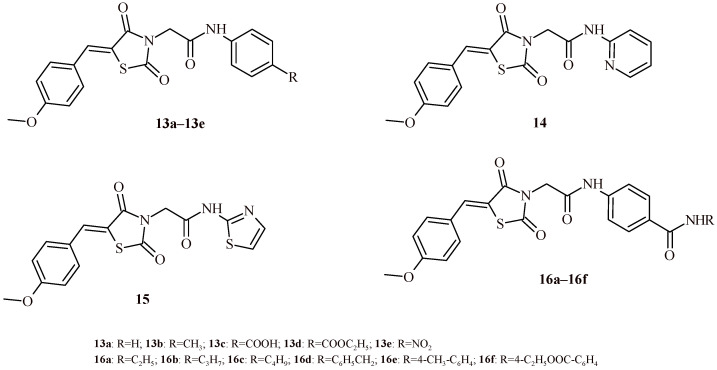
The 5-(4-Methoxybenzylidene)thiazolidin-2,4-dione Derivatives **13a**–**13e**, **14**, **15** and **16a**–**16f** with inhibitory effect on VEGFR-2.

The 5-(4-Methoxybenzylidene)thiazolidin-2,4-dione derivatives (**13a**–**13e**, **14**, **15** and **16a**–**16f**) were tested for their activity against the HepG2, HCT116 and MCF-7 cell lines [30,31]. Among all tested derivatives (Figure 8), Compounds **16f**, **16e**, **16d** and **16c** showed the highest antiproliferative activity in the in vitro studies against HepG2, HCT116 and MCF-7 cell lines. Their IC_50_ values were in the range of 5.1–22.08 µM. Compound **16f** showed most potent activity. It inhibited proliferation of HepG2 (IC_50_ = 6.19 ± 0.50 µM) and MCF-7 (IC_50_ = 5.10 ± 0.40 µM) cells better than reference drugs sorafenib (IC_50_ = 9.18 ± 0.60 µM for HepG2 and IC_50_ = 7.26 ± 0.30 µM for MCF-7) and doxorubicin (IC_50_ = 7.94 ± 0.60 µM and IC_50_ = 6.75 ± 0.40 µM for HepG2 and MCF-7, respectively). Derivative **16f** exhibited also activity against HCT116 comparable to doxorubicin (8.37 ± 0.70 µM vs. 8.07 ± 0.80 µM). All the obtained derivatives were tested for inhibitory activity against the vascular endothelial growth factor receptor-2 (VEGFR-2). Among them Compound **16f** exhibited most potent inhibitory activity with IC_50_ value 0.12 ± 0.02 µM that was comparable with results for reference drug sorafenib (IC_50_ = 0.10 ± 0.02 µM). Moreover, Compounds **16e**, **16d**, **16c** and **16b** showed also high potential towards VEGFR-2 with IC_50_ values 0.13 ± 0.02, 0.14 ± 0.02, 0.14 ± 0.02 and 0.18 ± 0.03 µM, correspondingly.

The next step of assay was molecular docking studies that was performed to investigate binding mode and affinities of compounds towards VEGFR-2. The docking studies performed in the Molsoft software showed that all derivatives assume a similar position and orientation at the receptor binding site. The proposed model of connection takes into account the affinity of the Derivative **16f** with a value of −103.50 kcal/mol and the formation of six hydrogen bonds. The carbonyl group at the position of the second thiazolidine-2,4-dione derivative joins forms one of these bonds with Asp1044. In addition, the NH group of the carboxamide linker forms another bond with Glu915. The oxygen atom from the carboxyl group stabilized the hydrogen bonds formed with Arg1025 and Ile1023. The 4-methoxybenzylidene substituent located in the hydrophobic cavity was formed by Arg1025, His1024, Ile1023, Cys1022, Leu1017, Ile890 and Ile886. Furthermore, the thiazolidin-2,4-one moiety itself occupies the hydrophobic cavity formed by Asp1044, Ile890, Leu887, Ile886 and Glu883. The central phenyl group is attached to the cavity provided by Leu1033, Cys917, Phe916, Leu838, and Ala864. The distant ethyl group, on the other hand, combines with the hydrophobic cleft formed by Gly920, Phe919, Lys918, Phe916 and Leu838. The described interactions help to understand the nature of such a strong anti-cancer effect of the **16f** derivative.

Molecular docking studies carried out for the remaining derivatives showed that the acetamide linker occupies the same cavity as the urea linker contained in the sorafenib structure. This plays a key role in increasing the affinity for the VEGFR-2 enzyme. The 4-methoxybenzylidene derivative compensates for the *N*-methylpicolinamide substituent effect of sorafenib and increase the chance for hydrogen bond formation as well as increase similarity towards the VEGFR-2 enzyme. The tiazolidine-2,4-dione core enables new compounds to form new hydrogen bonds via the carbonyl group at Position 2 with the basic amino acid Asp1044. Structure elongation plays an important role in the inhibition of VEGFR-2. Hydrophobic distal substituents and their connections give the chance for hydrogen bonds to be formed with the amino acid Glu915, which further increases the similarity to the VEGFR-2 enzyme. Worth noticing that the results of molecular docking studies are correlated very well with biological screening results. The obtained results show the potential usefulness of the considered compounds for the future design, optimization, adaptation and research in order to produce more potent and selective inhibitors of VEGFR-2 with higher anti-cancer analogues.

Tyrosinase is a catalyst that regulates the duration of the melanin synthesis process. Melanin is synthesized from l-tyrosine in melanosomes, where this process is controlled by many factors, including tyrosinase [32].

**Figure 9:**
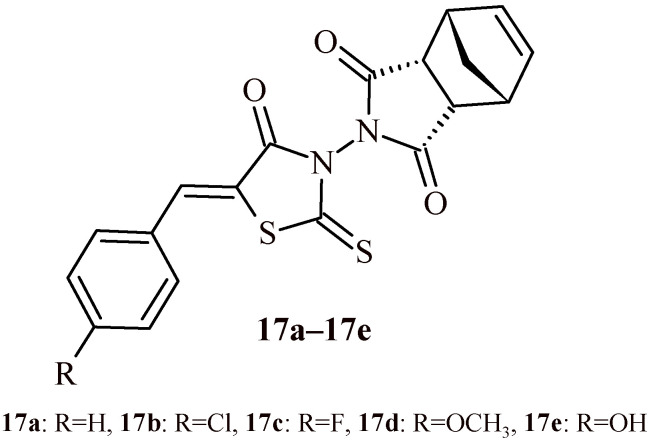
Rhodanine derivatives that inhibited melanogenesis.

Studies carried out by Isogawa et al. showed that the rhodanine Derivative **17c** (with 4-fluorobenzylidene substituent) strongly inhibited the melanogenesis process in mouse melanoma B16F10 cells in 10 µM concentration (Figure 9) [33]. Compound **17c** reduced the level of tyrosinase activity without modifying its messenger RNA levels or enzymatic activity. This derivative may promote the degradation of tyrosinase proteins; however, this degradation may be associated with simultaneous protein synthesis. Taking this into account, it was found that the Compound **17a** also lowered the activity level of the tyrosinase proteins, while having no effect on tyrosinase related protein 1 (TYRP-1), another protein involved in the melanogenesis process. Compound **17a** (with benzylidene substituent) promotes the breakdown of tyrosinase from TYRP-1 in B16F10 cells. In injured cells, tyrosinase is localized together with TYRP-1 in two regions—the peripheral and the nuclear. The Compound **17a** amplified the peri-nuclear signals while reducing the frequency of the peripheral signals. Other rhodanine Derivatives **17b** (with 4-chlorobenzylidene substituent), **17d** (with 4-methoxybenzylidene substituent) and **17e** (with 4-hydroxybenzylidene substituent) were less effective than Compound **17c**.

Considering the chemical structure, it can be seen that the phenyl group is the factor which determines the activity of the compounds. The phenyl group should not be modified (Compound **17a**) to preserve the stability of the compounds. Substitution with a fluorine (**17c**) (but not chloro—**17b**) atom in the *para* position of phenyl group is acceptable since this does not affect the activity of the compound. Compounds (**17a**–**17e**) were also analyzed for their hydrophobicity. No correlation was found between hydrophobicity and their potency.

Breast cancer is the most common cancer in women and ranks second in the number of deaths in women [34]. The demand for drugs to combat this cancer is incredibly high. Due to the wide spectrum of activity of thiazolidin-2,4-one derivatives, there are several reports about the breast cancer activity of these compounds.

**Figure 10:**
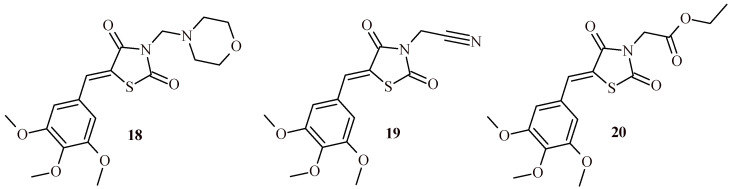
The 5-(3,4,5-trimethoxybenzylidene)thiazolidine-2,4-dione derivatives with anti-breast cancer activity.

In a 2020, El-Kashef et al. synthesized series of TZD derivatives with 5-(3,4,5-trimethoxybenzylidene) moiety (Figure 10). The synthesized TZDs were tested for their anti-breast cancer activity against human breast cancer cells (MCF-7 and MDA-MB-231) and also against normal non-cancerous breast cells that were obtained from the same patients [35]. Initial screening studies showed that Compounds **18**, **19** and **20** had the greatest anticancer activity. These derivatives inhibited the proliferation of breast cancer cells in a dose-dependent manner with an IC_50_ of 1.27, 1.50 and 1.31μM respectively. Using flow cytometric analysis, it was found that these three compounds mediated apoptosis of human breast cancer cells without affecting the survival of normal non-cancerous breast cells that had been isolated from the same patients. These compounds (**18**, **19** and **20**) strongly inhibited the proliferation of MCF-7 breast cancer cells by reducing the phosphorylation of AKT, mTOR and expression of VEGF and HIF-1α.

**Figure 11:**
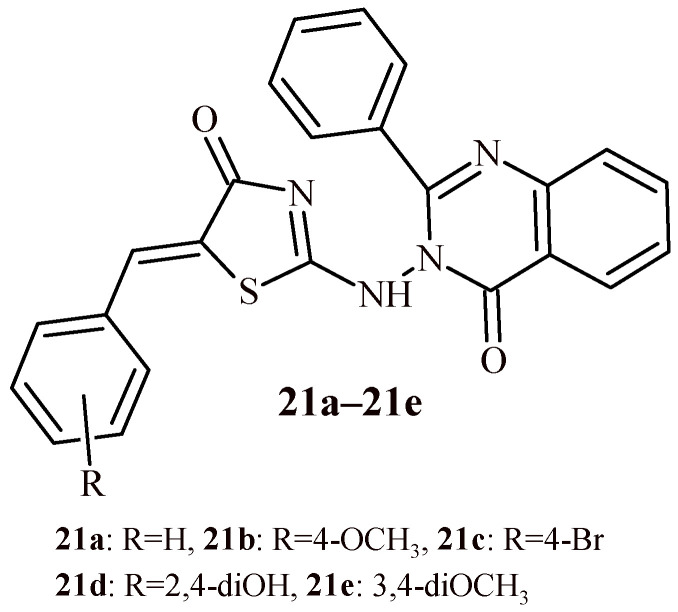
The structures of thiazolidin-4-one derivatives with EGFR inhibitory activity.

Aziz et al. conducted the synthesis of series quinazolinone-thiazolidin-4-one hybrids (**21a**–**21e**) and evaluated their anticancer activity against breast cancer (MCF-7) and lung cancer (A549) cell lines (Figure 11). Additionally, EGFR inhibitory activity of synthesized compounds was carried out [36].

Compounds **21a**–**21d** showed significant cytotoxic effect against both MCF-7 and A549 cell lines (Table 1).

However, Compound **21e** with electron donating groups (3,4-dimethoxy substituted derivative) was most active against MCF-7 cell line with IC_50_ value of 1.003 µM. It is worth noticing that unsubstituted Compound **21a** showed highest cytotoxic activity against A549 cell line with IC_50_ = 0.72 µM and more EGFR inhibitory activity at concentration 65.63 nM. Moreover, Compound **21a** exhibited good normal cell cytotoxicity profile (IC_50_ = 44.34 µM).

**Figure 12:**
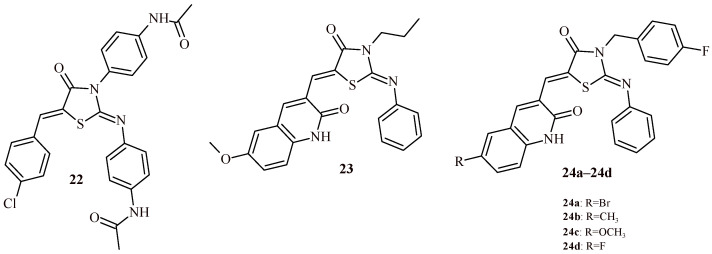
The structures of 2-iminothiazolidin-4-one derivatives with anti-breast cancer activity.

Abumelha and Saeed synthesized series of 5-arylidene-2-(4-acetamidophenylimino)thiazolidine-4-one derivatives and carried out their evaluation for anti-breast cancer activity (MCF-7) with use of MTT assay. Among series of ten compounds most active was Compound **22** (Figure 12). It showed half maximal inhibitory concentration at 58.33 µM. As a standard doxorubicin (IC_50_ = 48.06 µM) was used [37].

Kumar et al. conducted evaluation of anti-breast cancer activity of series of quinolinone-thiazolidin-4-one hybrids with use of MTT test on MCF-7 and MDA-MB-231 cell lines. As a result of the study, it was found that Compounds **23**, **24a**–**24d** were most potent in the series. Compound **24b** showed the best activity against MDA-MB-231 cell line (IC_50_ = 8.16 µM), meanwhile Compound **24c** was most effective against MCF-7 cancer cells (IC_50_ = 18.03 µM) (Table 2). It is worth noticing that Compounds **23** and **24a**–**24d** had low cytotoxicity towards HEK-293 cells [38].

**Figure 13:**
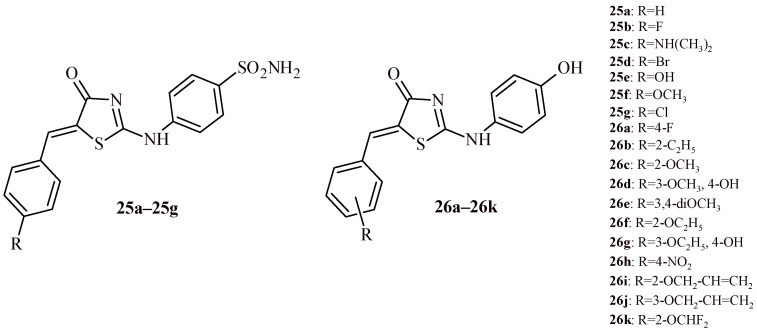
The structures of 5-arylidene-2-phenylaminothiazol-4(*5H*)-one derivatives with antiproliferative activity.

The series of Compounds **25a**–**25g** was synthesized by Nissan et al. (Figure 13). These derivatives were assessed against colon (HCT-116), lung (A549), breast (MCF-7) and liver (HepG2) cancer cell lines with use of sulforhodamine B method. For the colon and lung cancer cell lines none of the tested compounds exhibited equal or better activity that standard drug doxorubicin (IC_50_ were 0.058 and 0.27 µM, respectively). The Compounds **25a**–**25g** were active at half maximal inhibitory concentration in the range of 8.91–58.66 µM (HCT-116) and 11.73–60.47 µM (A549). Compound **25e** was most effective from series towards colon and lung cancer cell lines (IC_50_ = 8.91 and 11.73 µM). The Compounds **25b** and **25c** were between 4.5 to 2.7-fold more active towards breast cancer cells (MCF-7) than doxorubicin (IC_50_ = 1.50 µM). In addition, Compounds **25b**–**25d** showed high cytotoxicity against HepG2 cell line with IC_50_ values of 1.58, 0.24 and 2.28 µM, respectively. Compound **25c** was 3.7-fold more effective than doxorubicin (IC_50_ = 0.90 µM), whereas Derivative **25e** was weakly active (IC_50_ > 100 µM). Additionally, the most potent Compounds **25b**, **25c** and **25e** exhibited low cytotoxicity against normal breast cell line MCF-10A, showing good selectivity; SI > 270, > 185 and > 5.8, respectively [39].

In the next study the evaluation of 5-arylidene-2-phenylaminothiazol-4(*5H*)-ones against ten leukemia cell lines (four Ba/F3 cell lines, KBM7-SLF N233, KBM7_WT, Dami, HL-60, Jurkat and K-562) was presented. The Compounds **26a**–**26k** showed antiproliferative activity in submicromolar concentration at least for one cell line. The Compound **26c** showed strong cytotoxic activity against three Ba/F3 cell lines at IC_50_ values in the range of 0.10–0.24 µM and against Dami cell line (IC_50_ = 0.87 µM). The Compound **26i**, with 2-allyloxy substituent, possessed the highest antileukemic activity against Dami, HL-60, Jurkat and K-562 cell lines at submicromolar concentration 0.95, 0.17, 0.10 and 0.18, respectively [40].

**Figure 14:**
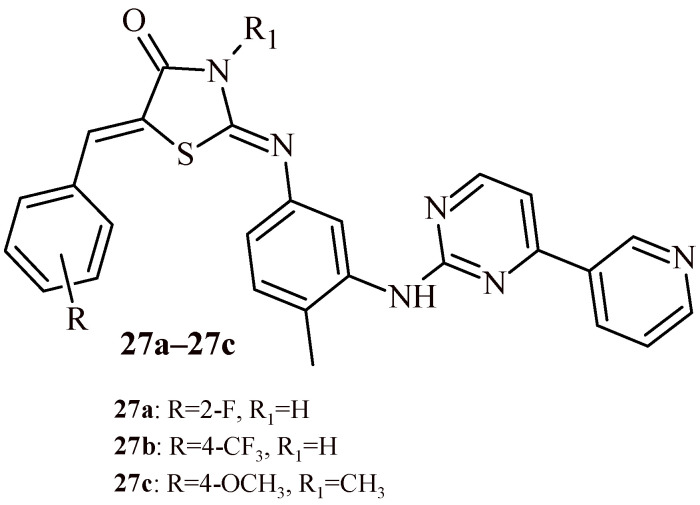
The thiazolidine-4-one-phenylaminopyrimidine hybrids with antileukemic activity.

Türe et al. described synthesis of thiazolidine-4-one-phenylaminopyrimidine hybrids and tested them for their anticancer activity against chronic myeloid leukemia (K-562), prostate cancer (PC3) and neuroblastoma (SHSY-5Y) cells. Only three compounds (**27a**–**27c**) among the series of 42 hybrids showed cell viability% values less than 50.0% on K562 cells (Figure 14). The IC_50_ against K-562 cell line was also determined for these three compounds. The IC_50_ was 8.79, 4.86 and 9.97 µM, respectively for Compounds **27a**, **27b** and **27c**. Additionally apoptosis studies, cell cycle experiments were carried out for **27a**–**27c**. As a results, **27a** and **27b** cause mainly early and late apoptosis in K-562 cells in a time-dependent manner. In the cell cycle assay, Compounds **27b** and **27c** arrest G0/G1 phase similar to imatinib (standard), **27a** induces cell cycle arrest G2/M phase [41].

**Figure 15:**
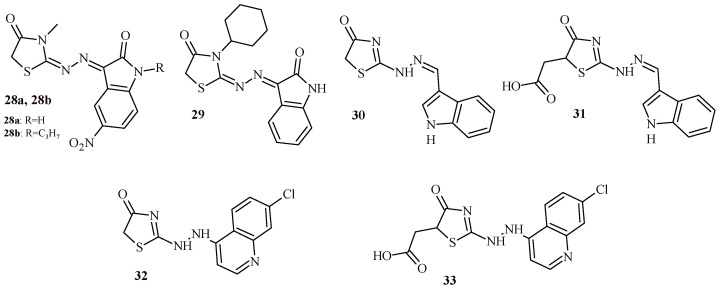
The structures thiazolidin-4-one-based indole and quinoline hybrids.

The series of isatin-based thiazolidin-4-one derivatives was synthesized and evaluated for their anticancer activity against three cancer cell lines (HepG2, MCF-7 and HT-29). The best activities among the series were found for Compounds **28a** and **28b** (Figure 15). The Compound **28a** was active against HepG2, MCF-7 and HT-29 at half maximal inhibitory concentration of 27.59, 8.97 and 5.42 µM, respectively. While Compound **28b** showed comparable activities to standard doxorubicin against all three cancer cell lines. The IC_50_ were 4.97 vs. 4.50 µM (HepG2), 5.33 vs. 4.17 µM (MCF-7) and 3.29 vs. 4.01 µM (HT-29). Moreover, these compounds showed satisfactory cytotoxicity against fibroblasts (WI-38) at concentration 98.22 µM (**28a**) and >100 µM (**28b**). Additionally, Compounds **28a** and **28b** were tested on inhibitory activity against CDK1, p53, caspase-3 and caspase-9. Compounds **28a** and **28b** exhibited comparable or more potent activity than doxorubicin used as standard (Table 3) [42].

Fouad et al. reported anticancer activity of isatin-based thiazolidin-4-one **29** against renal cell carcinoma. Compound **29** showed growth inhibition % of all tested five renal cancer cell lines in the range 59.69–78.46%. The values of half maximal inhibitory concentration were 8.1 µM (A498), 17.47 µM (ACHN), 4.74 µM (CAKI-1), 23.57 µM (RXF393) and 3.99 µM (UO-31). Moreover, Compound **29** showed comparable activity to reference drug sunitinib (IC_50_ = 5.51 µM) against CAKI-1 renal cancer cell line. In addition, selectivity index of tested compound toward normal renal cell line against five renal cancer lines was in the range of 1.23–7.24. Assays of activity of Compound **29** were performed against angiogenesis promoting enzymes: VEGFR-2, PDGFRα and PDGFRβ. The IC_50_ values were at submicromolar concentration 452.53 nM (VEGFR-2), 280.916 nM (PDGFRα) and 45.013 nM (PDGFRβ). In the case of PDGFRβ, enzyme activity was better than sunitinib (IC_50_ = 55 nM) [43].

Whereas thiazolidin-4-one derivatives with indole (**30**, **31**) and quinoline (**32**, **33**) moieties showed weak antiproliferative effect against SF-295, HL-60 and PC3 cell lines. Their reduction of cell viability was less than 40.8% [44].

**Figure 16:**
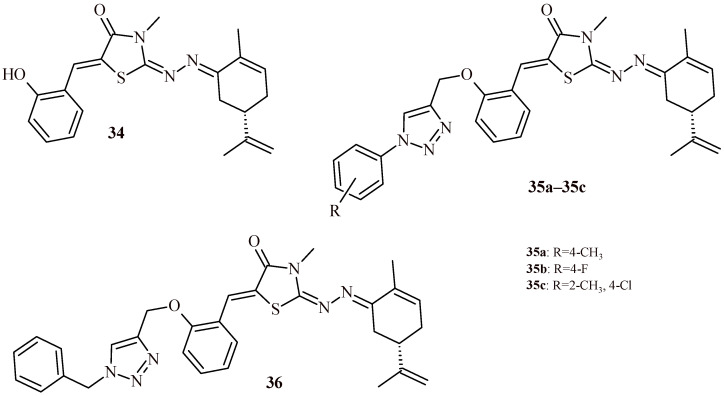
The thiazolidin-4-one carvone hybrids as anticancer agents.

Oubella et al. described synthesis and evaluation of carvone-thiazolidin-4-one hybrids with 1,2,3-triazole moiety (**34**–**36**) for their anticancer activity against HT-1080, A-549, MCF-7 and MDA-MB-231 cell lines. As a result of the study, it was found that Compounds **35b** and **35c** (Figure 16) exhibited the most potent cytotoxic activity against HT-1080 and A-549 cell lines (Table 4). In the next step of the study, the cell death pathway of the cytotoxic activity of **35b** and **35c** was investigated. The results suggested that they are capable of inducing apoptosis in a caspase-3 pathway manner and to affecting the cell cycle [45].

**Figure 17:**
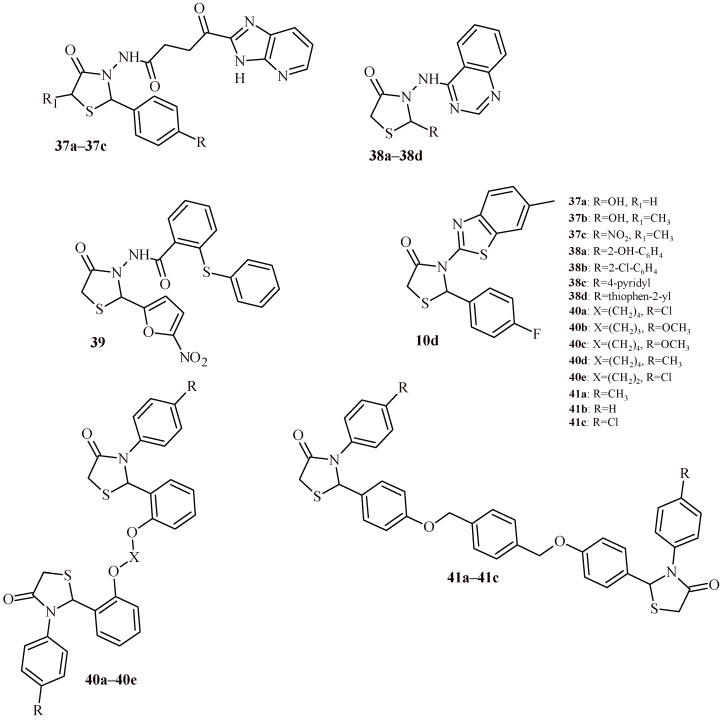
The 2-Aryl-3-aryl/arylaminothiazolidin-4-one derivatives with anticancer activity.

Iqbal and co-worker reported anticancer activity for Compounds **37a**–**37c** as most effective among the series of imidazopyridine-thiazolidin-4-one hybrids (Figure 17). The Compound **37a** with *para*-hydroxy group was found most potent against MCF-7 and DU145 with IC_50_ values 3.2 and 6.8 µM, respectively. Whereas Compound **37b** (5-methyl analogue of **37a**) was most effective against A549 (IC_50_ = 8.4 µM). The nitro substituted derivative (**37c**) showed slightly less activity than **37b** against A549 (IC_50_ = 9.9 µM). Worth noticing that Compounds **37a** and **37b** with *para*-hydroxy group showed additionally antioxidant activity in DPPH assay (EC_50_ values of 40.26 and 39.72 µM, correspondingly) comparable with ascorbic acid used as standard (EC_50_ = 35.62 µM) [46].

The introducing of quinazoline moiety in Position 3 of thiazolidin-4-one ring decreased cytotoxic activity against A549 and MDA-MB-231 cell lines. The Compounds **38a**–**38d** showed weak cytotoxic effect at concentration in the range of 134.77–153.20 µg/mL [47].

The thiazolidine-4-one derivative (**39**) with 5-nitrofuran-2-yl substituent exhibited promising anti-breast cancer activity in the in vitro and in vivo tests. The IC_50_ values for anticancer activity of Compound **39** against human cancer cell lines were 1.9 µM (MDA-MB-231), 5.4 µM (HepG2), 6.5 µM (HT-29) and 13.51 µM towards normal cell line HGF-1, after treatment for 72 h. In order to establish the mechanism of action of the Compound **39**, it carried out series of the tests: induction of cell apoptosis, evaluating the cell cycle progression promoted anchorage-independent growth. In vivo tests were carried out with use of orthotopic breast cancer tumor model on female BALB/c mice. The Compound **39** at Concentration 1 and 10 mg/kg showed 33% and 66% reduction in the tumor growth at termination of experiment. Additionally, it was conducted an assay of Compound **39** on suppress of the metastasis lung. The results showed that Compound **39** contributed to its reduction (39.8% vs. 59.8 in the control group) confirming anti-metastatic effect of Compound **39**. The potential activity of Compound **39** is most likely due to induction of apoptosis with G1/S arrest as well as inhibition of angiogenesis [48].

Besides the previously described antioxidant properties of Compound **10d** (Figure 5 and Figure 17), this derivative demonstrated dose dependent anticancer activity against Ehrlich ascites carcinoma (EAC) induced peritoneal ascites in mice. The outcomes were comparable to standard 5-fluorouracil [23].

Hebishy et al. presented results of cytotoxic activity of bis-thiazolidin-4-ones (**40a**–**40e** and **41a**–**41c**). In Table 5, results of compounds are presented. It shows the best activity of them against HepG2, MCF-7 and Caco-2. Compound **40a** was the most effective against MCF-7 and Caco-2 cell lines with IC_50_ values of 22.04 and 45.91 µM, respectively. The Compound **40b** was the most active against HepG2 cells (IC_50_ = 34.94 µM). Generally, *ortho* substituted derivatives (**40a**–**40e**) exhibited better cytotoxic activity than *para* derivatives (**41a**–**41c**) [49].

**Figure 18:**
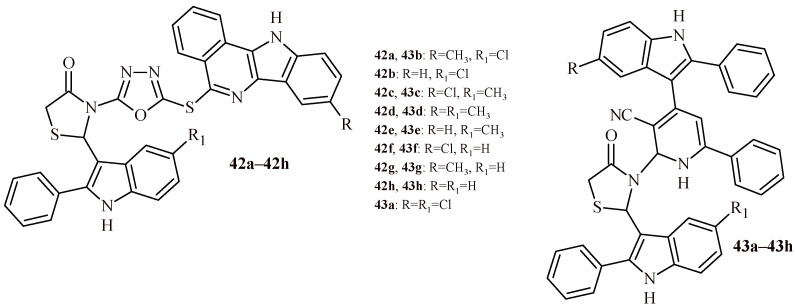
The structures of a series of thiazolidin-4-one-1,3,4-oxadiazoles (**42a**–**42h**) and thiazolidin-4-one with indolyl-pyridine moiety (**43a**–**43h**).

Verma et al. synthesized series of thiazolidin-4-one-1,3,4-oxadiazoles (**11**, **42a**–**42h**) and tested them for anticancer activity against MCF-7, A549 and HeLa cell lines (Figure 7 and Figure 18). The Compound **42d** showed noteworthy cytotoxic activity against all three cell lines, MCF-7 (IC_50_ = 0.47 µM), A549 (IC_50_ = 0.59 µM) and HeLa (IC_50_ = 0.53 µM). These values were comparable to doxorubicin (0.58, 0.72 and 0.89 µM, respectively).

The Compounds **42g** and **42h** also expressed enhanced activity at half maximal inhibitory concentration of 1.54 and 1.82 µM (MCF-7), 1.78 and 1.85 µM (A549), 1.56 and 1.80 µM (HeLa). The other compounds showed moderate effect (**42a**–**42c**) or were inactive (Compound **11**, Figure 7) [25]. Similar results were obtained also for series of Compound **12** and **43a**–**43h** (Figure 7 and Figure 18). The most effective Derivative **43d** showed activity against three cell lines at concentration 0.45 µM (MCF-7), 0.53 µM (A549) and 0.52 µM (HeLa) [26].

**Figure 19:**
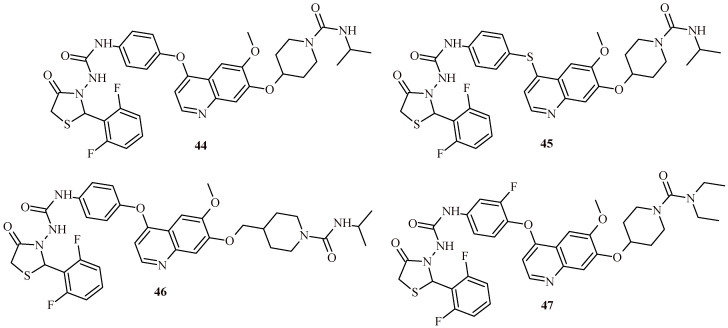
The structures of quinoline-based 2-(2,6-difluorophenyl)-thiazolidin-4-one derivatives as kinase inhibitors for the treatment of colorectal cancer.

Zhou et al. described assessing of the quinoline-based 2-(2,6-difluorophenyl)-thiazolidin-4-one derivatives as kinase inhibitors for the treatment of colorectal cancer [50,51]. Among the fifteen derivatives, identified the most active Compound **44** showed cytotoxic activity against HT-29 cell line at IC_50_ value 0.31 ± 0.022 µM (Figure 19). Furthermore, **44** inhibited tyrosine-protein kinase Met (c-Met) and Ron tyrosine kinase in 65 and 84.4% with IC_50_ values 0.382 and 0.122 µM, respectively. The thio analogue of **44**, Compound **45**, showed slightly less activity against HT-29 cells (IC_50_ = 0.42 ± 0.031 µM), while maintaining a similar level of kinases inhibition (62.9%—c-Met; 83%—Ron). The homologue of **44**, Compound **46**, exhibited similar level of kinases inhibition (58.4% —c-Met; 86.1% —Ron) and cytotoxic effect against HT-29 cells (IC_50_ = 0.39 ± 0.028 µM). These results were more potent than same for standard drugs regorafenib (IC_50_ = 2.87 ± 0.18 µM against HT-29) and cabozantinib (IC_50_ = 10.6 ± 1.12 µM against HT-29) [50].

The modification of ureidophenoxyl and piperidine-1-carboxamide moieties by 2-fluoro and diethyl substituents led to obtaining more effective (Compound **47**) anticolorectal cancer agent than Compound **44**. The IC_50_ value of **47** was 0.19 ± 0.012 µM, % of inhibition was 76.3 and 89.3 against c-Met and Ron kinases [51].

**Figure 20:**
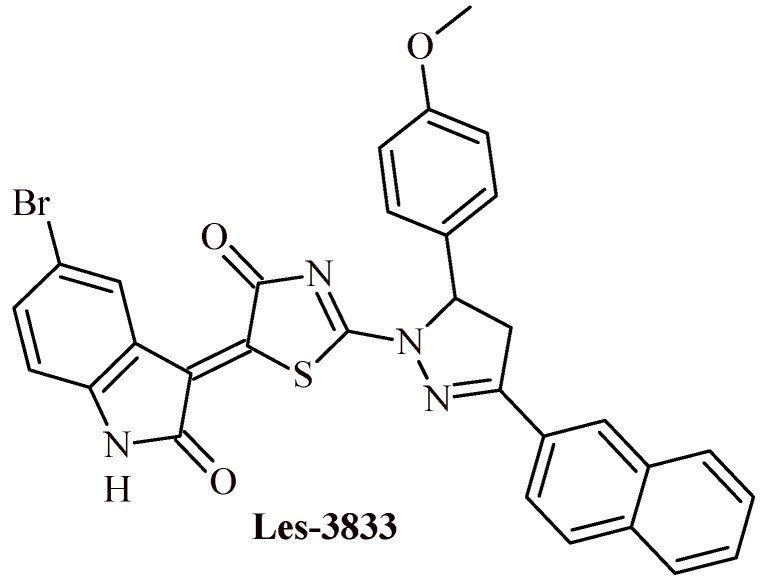
The structures of thiazolidin-4-one-based derivative Les-3833.

Kobylinska et al. reported anticancer activity of Les-3833 comparable to doxorubicin against human glioma and rat glioma cells as well as human melanoma cell lines (Figure 20). Their IC_50_ were 0.84 ± 0.09 (Les-3833) vs. 0.90 ± 0.11 (doxorubicin) µg/mL (U251), 0.89 ± 0.12 vs. 0.84 ± 0.08 µg/mL (C6), 0.22 ± 0.03 vs. 0.24 ± 0.05 µg/mL (WM793) and 0.30 ± 0.04 vs. 0.35 ± 0.06 µg/mL (SK-MEL-28) [52]. Moreover, in silico studies of Les-3833 showed the affinity to topoisomerase II inhibitor, and high possibility for inhibitory interaction with ChekPoint kinase-1, caspase-6 and caspase-8 [53].

**Figure 21:**
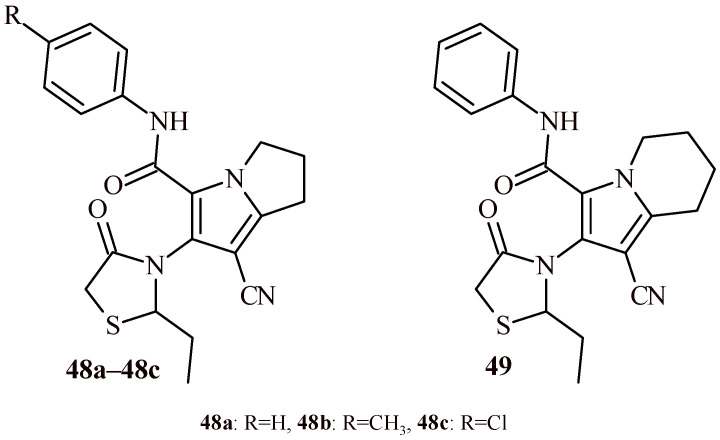
Pyrrolizine-thiazolidin-4-one derivatives with anticancer activity.

The studies conducted by Shawky et al. concern to anticancer activity of pyrrolizine-thiazolidin-4-one derivatives (**48a**–**48c** and **49**) (Figure 21) [54]. The cytotoxicity was evaluated by the MTT assay. The results displayed potential cytotoxic activity with IC_50_ values in the range of 0.11–4.24 µM, toward three cancer cell lines (MCF-7, A2780 and HT-29). Among the pyrrolizine-thiazolidin-4-one hybrids (**48a**–**48c** and **49**), Compound **48a** showed highest anticancer potential against breast cancer cell line (MCF-7) with IC_50_ = 0.16 ± 0.08 µM. While Compound **48b** was the most active against A2780 (IC_50_ = 0.11 ± 0.01 µM) and HT-29 (IC_50_ = 0.12 ± 0.03 µM). The Compounds **48a**–**48c** and **49** were also evaluated for their selectivity toward normal MRC5 cells. Generally, pyrrolizine-thiazolidin-4-one derivatives exhibited selectivity index (SI) in the range of 3–15 against MCF-7 and A2780 cell lines. The Compound **49** showed the highest selectivity against all three cell lines (SI = 2.62 for A2780 cells, SI = 5.39 for HT-29 and SI = 13.21 for MCF-7cells).

**Figure 22:**
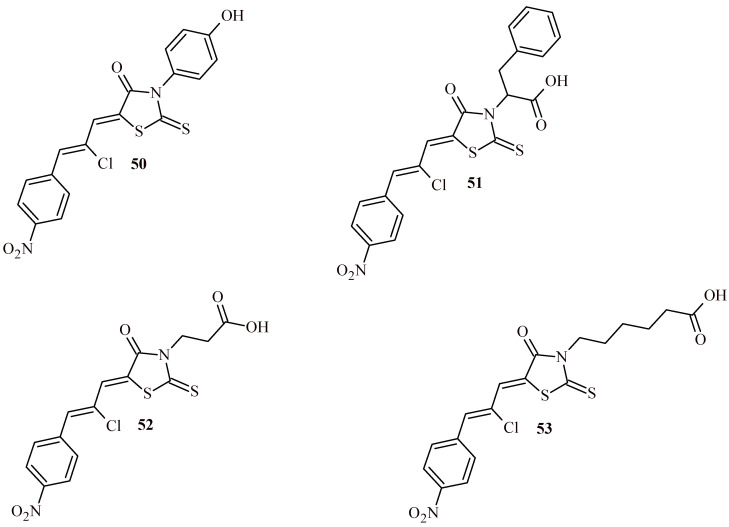
The ciminalum-rhodanine hybrids with anticancer properties.

Lesyk et al. synthesized series of ciminalum-rhodanine hybrids and conducted screening of obtained compounds for their antitumor activity according to the NCI DTP (USA) standard protocol at the concentration in the range 10^−4^–10^−8^ M against 60 tumor cell lines [55]. The results of screening of ciminalum-rhodanine hybrids confirmed their significant anticancer properties. Compounds **50** and **52** (Figure 22) showed growth inhibition of all tested tumor cell lines at submicromolar and micromolar concentrations. Worth noticing that the most active Compound **52** was active with GI_50_ concentration in the range of <0.01–0.02 µM against following cell lines: MOLT-4, SR (leukemia), SW-620 (colon cancer), SF-539 (CNS cancer), SK-MEL-5 (melanoma). Based on SAR analysis, authors note that the presence of ciminalum moiety turned out to be essential for achievement of the anticancer activity. Furthermore, nature of substituents at Position 3 of thiazolidinone ring is important. Thus, compounds with 4-hydroxyphenyl substituent (**50**) and carboxylic acid residue (**51**, **52**) were most active against cancer cell lines.

In the next stage of the research, obtained hybrids were evaluated for their anticancer activity against AGS (gastric cancer), DLD-1 (human colon cancer), MCF-7 and MDA-MB-231 (breast cancers) by MTT assay. The most effective were Compounds **52** and **53** with propanoic and hexanoic acids residue at Position 3 of rhodanine ring, respectively. Their GI_50_ values were as follows: for Compound **52**—2.69 µM (AGS), 3.67 µM (DLD-1), 3.62 µM (MCF-7) and 1.63 µM (MDA-MB-231); for Compound **53**—3.20 µM (AGS), 9.22 µM (DLD-1), 1.73 µM (MCF-7) and 0.95 µM (MDA-MB-231). These results were consistent with previously obtained data according to NCI DTP protocol for breast cancers cell lines. Moreover, Compounds **50**–**53** had low toxicity toward normal human blood lymphocytes (GI_50_ values of 48.97 µM (**51**), 54.54 µM (**52**) and ~100 µM (**50** and **53**)). Summarizing, the important role of the ciminalum substituent in Position 5 and the nature of substituent in Position 3 (preferable carboxylic acid residue) of the rhodanine ring for anticancer activity was established.

**Figure 23:**
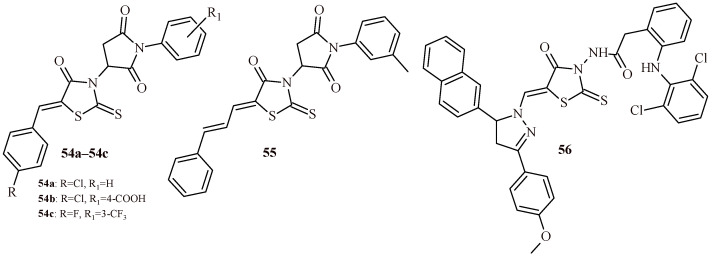
The structures of rhodanine-*N*-arylsuccinimid and rhodanine-pyrazoline hybrids.

In the next study the evaluation of cytotoxic potential of a series rhodanine-*N*-arylsuccinimid hybrids (19 compounds) against four leukemic cell lines (Dami, HL-60, Jurkat and K-562) was conducted [56]. Among the all-tested compounds, only Derivatives **54a**–**54c** and **55** (Figure 23) showed % of growth inhibition more than 50% at concentration 10 µM against Dami cell line.

The study conducted by Shepeta et al. showed that rhodanine-pyrazoline hybrid with diclofenac moiety (**56**) had high effectiveness against 60 cancer cell lines (NCI DTP protocol) with a mean of GI_50_ = 0.71/1.09 µM and TGI = 82.95/28.46 µM [57].

**Figure 24:**
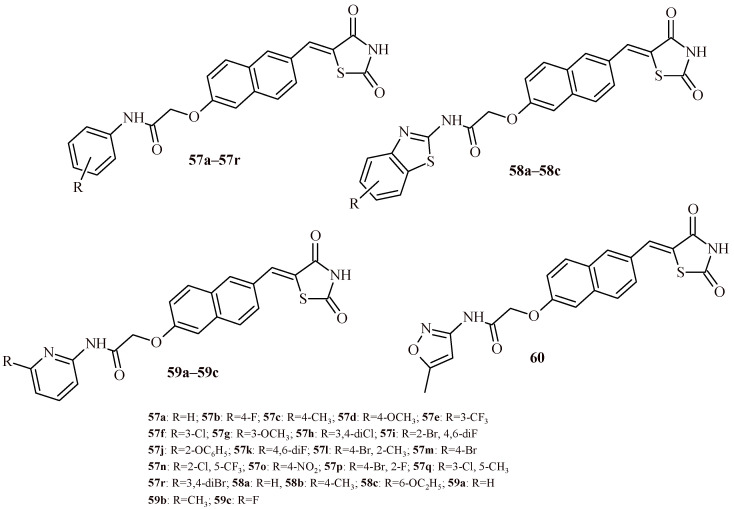
The structures of thiazolidine-2,4-dione derivatives with PPARγ and HDAC target simultaneously.

Tilekar et al. described series of 5-(naphtha-2-ylidene)thiazolidine-2,4-dione derivatives as simultaneously target PPARγ and HDAC (Figure 24). The reports [58,59,60] suggested that combination treatment with PPARγ agonists and HDAC inhibition increased cytotoxic activity against various cancer cell lines. Basing on these observations, authors designed, synthesized assessed of compounds that can be target PPARγ and HDAC simultaneously. Among the 25 compounds (**57**–**60**), several of them inhibited HDAC4 (**57l**, **58a** and **59c**) and six compounds (**57c**, **57i**, **57l**, **57m**, **57o** and **57p**) showed dual inhibition activity. Compound **58a** with benzothiazole moiety was most active against HDAC4 (IC_50_ = 0.42 ± 0.05 µM). Compound **58a** was the most effective also against HDAC8 (IC_50_ = 2.7 ± 0.2 µM). The most potent partial PPARγ agonist were **57i** and **57l** with EC_50_ values of 0.245 ± 0.006 µM and 0.359 ± 0.031 µM, correspondingly. The Compound **57i** was found to be most effective with dual activity (EC_50_ = 0.245 µM and IC_50_ = 1.1 µM). Moreover, Compounds **57c** and **57i** showed cytotoxicity against CCRF-CEM cells (CC_50_ = 2.8 and 9.6 µM, respectively), as well as they induced apoptosis and caused DNA fragmentation. Additionally, Compound **57c** caused in vivo tumor regression in CCRF-CEM tumor xenografts [61].

### 2.3. Anti-Inflammatory and Analgesic Properties

The above-mentioned Compounds **48a**–**48c** and **49** with anticancer activity were additionally tested for their anti-inflammatory activity as well as COX inhibitory activity (Figure 21) [54]. Compound **48c** showed only 21.76% inhibition of edema (2h post-carrageenan) and 32.48% inhibition of edema (4h post-carrageenan) it was worse than standard celecoxib (36.96% and 67.37%, respectively) and indomethacin (32.30% and 56.39%, respectively), whereas, high selectivity for COX-2 showed Compounds **48b** and **48c**. The Compounds **48b** and **48c** inhibited COX-2 with IC_50_ = 1.25 µM and 1.15 µM, correspondingly. The inhibitory activity of these both derivatives (**48b** and **48c**) towards COX-1 was >100 µM. Thus, their SI were >80 and >86, correspondingly.

**Figure 25:**
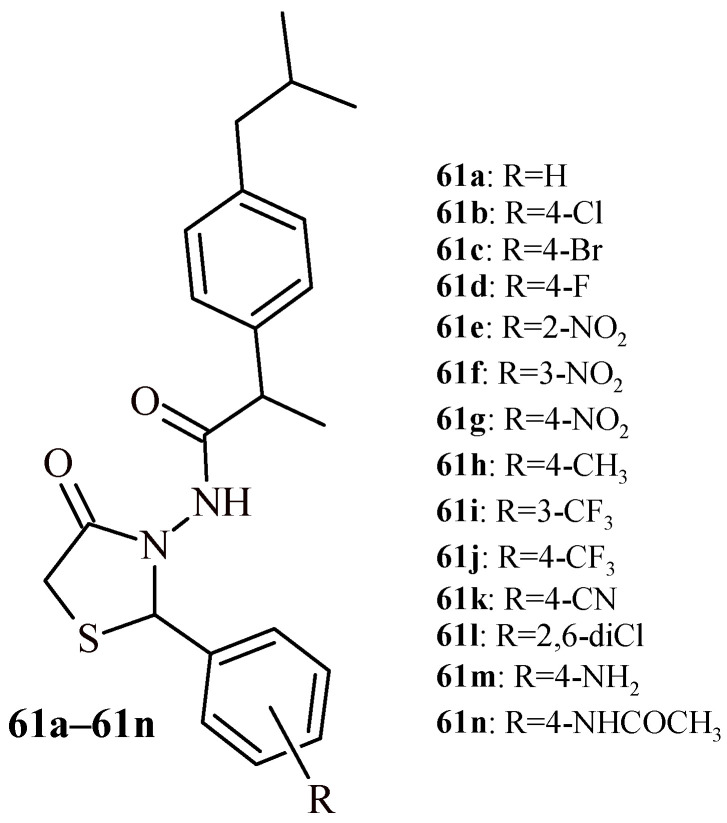
Ibuprofen-thiazolidin-4-one hybrids with anti-inflammatory and analgesic activities.

Vasincu et al. carried out evaluation of ibuprofen derivatives with thiazolidine-4-one scaffold (**61a**–**61n**) for their anti-inflammatory and analgesic activities (Figure 25) [62]. The carrageenan-induced paw edema assay was used to assess the anti-inflammatory effect. Additionally, the analgesic effect was verified by the tail-flick assay and the writhing test. First of all, acute toxicity on rat model was evaluated for all compounds (**61a**–**61n**). These compounds were less toxic than the reference drug—ibuprofen (LD_50_ = 1375 mg/kg b.w.) with LD_50_ values in the range of 1565–1840 mg/kg b.w. The results suggested that tested derivatives were practically non-toxic. The effects of the carrageenan-induced paw edema assay were measured at regular intervals (2 h, 4 h, 6 h and 24 h). The compounds were tested at a dose of 1/20 LD_50_ and the results are expressed as edema inhibition (%). At 6 h after administration, the most active compounds were **61d** and **61e**, the inhibition and percentages of paw edema were 65.71 ± 10.49% and 60.81 ± 8.49%, that were higher than ibuprofen value (43.67 ± 5.20%). Analysis of the data recorded 24 h after administration showed a long-lasting anti-inflammatory effect of the obtained compounds, for some even higher than that of reference drug ibuprofen. The most active of them was **61d** (with 4-fluorophenyl substituent), with an edema inhibition value of 53.04 ± 13.17%.

The highest analgesic activity in tail-flick assay showed Compounds **61m** (75.67 ± 5.94%), **61k** (75.36 ± 3.08%) and **61h** (74.45 ± 6.06%). The pain-inhibiting effects were higher than for ibuprofen (67.15 ± 8.66%). Among the tested compounds, the Derivatives **61n** (69.59 ± 5.56%), **61i** (68.37 ± 4.70%), **61e** (63.50 ± 6.49%) showed ibuprofen-like activity. The analgesic effect for the writhing assay was a measure of writhing episode number and it was expressed numerically. In this test, the activity of the synthesized derivatives was between 23.38 ± 1.45% to 56.37 ± 10.30%. For comparison, the registered value for ibuprofen was 52.37 ± 10.33%. The higher than the reference drug analgesic effect was demonstrated by the Compounds **61h** (56.37 ± 10.30%) and **61e** (53.06 ± 10.63%).

**Figure 26:**
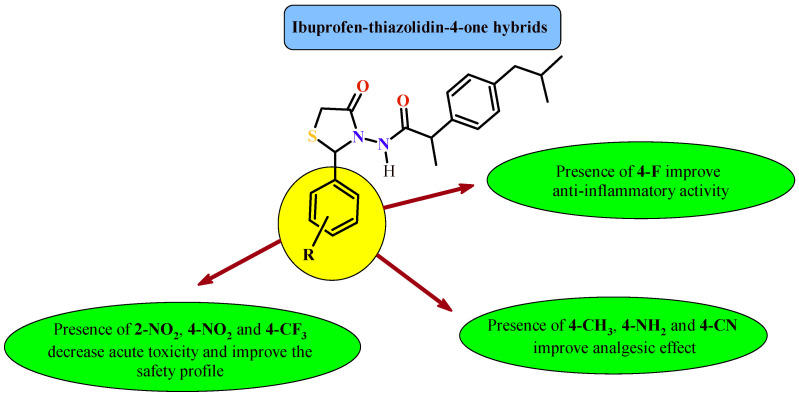
The influence of benzene ring substitution for activity potential of ibuprofen-thiazolidin-4-one hybrids.

The highest therapeutic potential was shown in compounds named **61d**, **61e**, **61k** and **61m**. It can be concluded that the presence of the trifluoromethyl group in the 4 position (**61j**) and the nitro group in the 2 or 4 positions (**61e** and **61g**) reduces toxicity and thus improves the safety profile of drugs. The compounds substituted with methyl (**61h**), cyano (**61k**), amino (**61m**) groups in the *para* position showed the strongest analgesic effect, but it was higher than activity of ibuprofen. The derivative which has a fluorine atom in its structure in the *para* position (**61d**) had the highest anti-inflammatory activity in the edema inhibition test (Figure 26).

**Figure 27:**
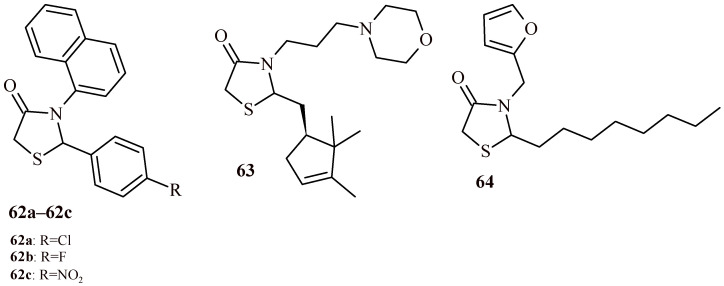
The structure of various 2-aryl/alkylthiazolidin-4-ones with potential anti-inflammatory and analgesic activity.

Among the 2-aryl-3-(naphtha-2-yl)thiazolidin-4-one derivatives described by Agrawal et al. the compounds (**62a**–**62c**) with chloro, fluoro and nitro substituents respectively showed most potent anti-inflammatory activity in carrageenan-induced paw edema test in rats (Figure 27). Besides, these derivatives were found to be the most potent also as analgesic agents in tail immersion and writhing tests in mice [63].

The Compound **63** with campholenic aldehyde residue in structure (Figure 27) at a dose of 100 mg/kg showed significant protection against indometacin-induced ulceration. It antiulcerative activity score (5.2) was higher than the same for reference drug omeprazole (3.8). Furthermore, Compounds **63** and **64** at a dose of 100 mg/kg had anti-inflammatory effects similar to standard diclofenac in histamine-induced inflammatory edema [64].

**Figure 28:**
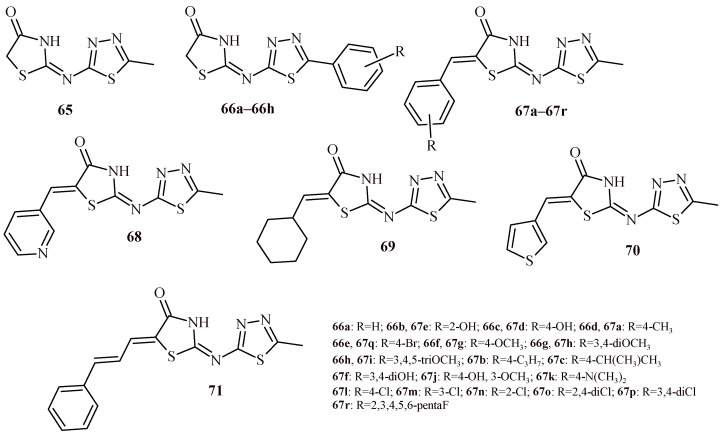
The structures of thiazolidin-4-one-1,3,4-thiadiazole hybrids with dual COX-2 and 15-LOX anti-inflammatory activity.

Omar et al. carried out evaluation of 31 thiazolidin-4-one-1,3,4-thiadiazole hybrids (**65**–**71**) for their activity towards 15-LOX and COX enzymes (Figure 28). Some of them were also tested for anti-inflammatory activity in vivo. The Compounds **65** and **66a** were most active against 15-LOX with IC_50_ values of 2.74 and 2.54 µM, respectively. In the arylidene series (**67a**–**67r**), Compound **67a** with 4-methyl group showed the highest activity (IC_50_ = 3.11 µM). Increasing the length of aliphatic substituent (**67b**, **67c**) decrease the activity (IC_50_ = 5.24 and 8.33 µM, respectively). The Compound **67e** with 2-hydroxy group was more active than 4-hydroxy isomer **67d** (IC_50_ values of 4.21 vs. 5.47 µM). Increasing the number of hydroxy groups decreased inhibitory activity (**67f**). Similar trends could be observed for derivatives with methoxy groups (**67g**, **67h** and **67i**) [65].

All tested compounds (**65**–**71**) exhibited inhibitory potential against COX-2 at concentration in the range of 0.1–0.42 µM and selectivity index ranging from 12.44 to 151.10. The high selective compounds were **67r**, **67i**, **67q** and **67f** with SI of 151.10, 123.40, 114.20 and 103.82, respectively. In addition, these derivatives showed higher activity against COX-2 than diclofenac. Basing on SAR analysis, authors determined that presence of bulky substituent (4-bromo or 3,4,5-trimethoxyphenyl) is required at Position 5 of thiazolidine ring for good activity and selectivity.

Selected compounds (**65**, **67f**, **67i**, **67q** and **67r**) were evaluated for their anti-inflammatory activity in vivo used carrageenan-induced paw edema model in rats. All the tested compounds at a dose 28 µg/kg showed maximal activity 3h after administration comparable to the reference drug celecoxib. The most active compounds (**65**, **67q** and **67r**) showed percent of inhibition of edema 49.5, 60.7and 57.9%, respectively [65].

**Figure 29:**
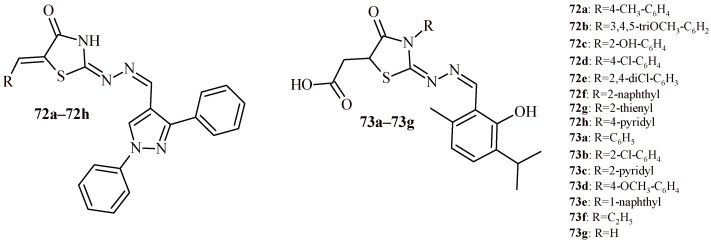
Thiazolidin-4-one derivatives containing pyrazole and thymol moieties with anti-inflammatory activity.

The series of 5-arylidenethiazolidin-4-ones with pirazole moiety was tested for their anti-inflammatory activity with use of carrageenan-induced rat paw edema test. Eight compounds (**72a**–**72h**) showed the most potent activity at the 4th h post administration (Figure 29). The percents of edema inhibition were 98.16% (**72g**), 96.73% (**72c**), 88.81% (**72d**), 81.5% (**72a**), 76.68% (**72e**), 76.17% (**72h**), 73.82% (**72f**) and 71.8% (**72b**). It is worth noticing, that Compound **72g** with 2-thienyl substituent showed higher edema inhibition percentage compared to all reference drugs, which were used in the test: indomethacin (96.94%), diclofenac (73.86%) and celecoxib (73.40%).

Selected derivatives were tested for their analgesic activity and the ability to inhibit the production of the inflammatory cytokine TNFα in serum. The results showed, that tested compounds (**72a**, **72c**, **72d** and **72h**), except **72d**, had rapid analgesia 30 min after administration as well as long analgesic effect for 90 min after administration. ELISA assay demonstrated that compounds (**72a**, **72c**, **72d** and **72h**) had ability to inhibit cytokine TNFα in mice sera in comparison reference drugs [66].

Presented in Figure 29 Compounds **73a**–**73g** showed multitarget inhibitory activity against COX-2, 5-LOX and PIM-1 kinase enzymes that are key for colorectal cancer treatment. The Compounds **73a**–**73d** showed high or equal activity and selectivity against COX-2 (IC_50_ = 0.037–0.058 µM; SI = 203.6–378) in comparison to reference drug celecoxib (IC_50_ = 0.045 µM; SI = 327). The most active against COX-2 as well as 5-LOX was Compound **72d** (IC_50_ values of 0.037 and 2.81 µM, respectively). Formalin-induced paw edema test indicated, that Compounds **73a**,**73f** and **73g** showed excellent anti-inflammatory activity compared to both reference drug diclofenac and celecoxib. Percents of edema inhibition were in the range of 53.90–72.23%. In vitro anticancer screening showed that Compounds **73d**, **73e** and **73g** were most active against human colon cancer cell lines (Caco-2 and HCT116) at ranges of concentration 0.051–0.114 and 0.06–0.109 µM, respectively. These compounds (**73d**, **73e** and **73g**) were tested to their ability to inhibit PIM-1/2 kinase enzymes. All compounds showed high inhibitory activity against PIM-1 and PIM-2 kinases. Compound **73g** was most effective against PIM-1 kinase (IC_50_ = 2.962 µM), and Compound **73d** was the most active against PIM-2 kinase (IC_50_ = 0.976 µM). The docking studies in the active site of target enzymes were supported by the biological results. In conclusion, Compound **73g** was found as effective inhibitor of three targets that lead to inhibition of human colorectal cancer cell proliferation [67].

**Figure 30:**
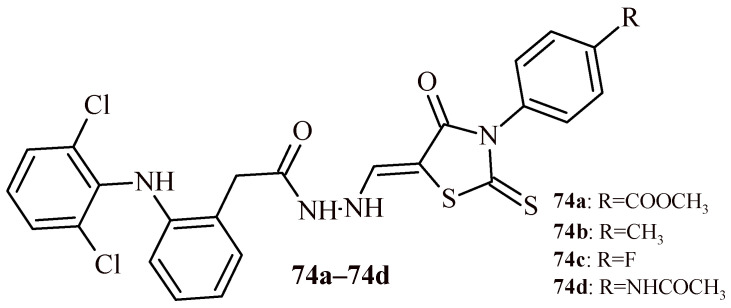
The rhodanine-diclofenac hybrids with anti-inflammatory activity.

There is also report of evaluation of anti-inflammatory activity of series rhodanine-diclofenac hybrids (Figure 30). Evaluation of anti-inflammatory activity, with use of carrageenan-induced rat paw edema test, indicated, that Compound **74b** showed no significant decreased of edema, whereas Compounds **74a**, **74c** and **74d** exhibited anti-inflammatory activity in the range of 39.5–40.8% of edema inhibition. It was comparable to reference drugs diclofenac and ketorolac. The most active was Compound **74c** with 4-fluorophenyl substituent in Position 3 of rhodanine [68].

### 2.4. Anticonvulsant Activity

**Figure 31:**
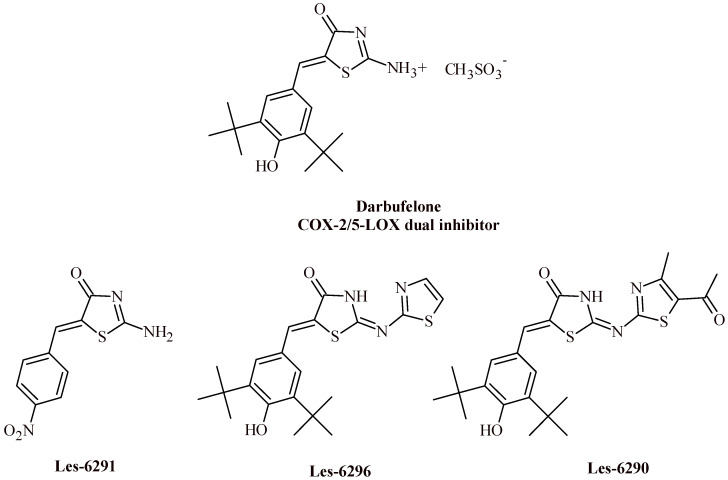
Darbufelone and its analogues with anticonvulsant activity.

Epilepsy is one of the most common brain diseases, affects over 70 million (1%) people worldwide [69]. The available protocols for the treatment of epilepsy are very often imperfect, therefore the research of new anticonvulsants are real problems for medicinal chemistry. Mishchenko et al. conducted synthesis of darbufelone analogues (Les-6290, Les-6291 and Les-6296) and evaluated their anticonvulsant effects in scPTZ test (Figure 31) [70]. As a reference drugs, darbufelone methansulfonate, celecoxib, sodium valproate and phenytoin was used in these experiments. Darbufelone methanosulfonate is a COX-2/5-LOX dual inhibitor with anti-inflammatory activity used in the treatment of rheumatoid arthritis [71,72]. All the obtained derivatives showed a good pharmacological effect in terms of anticonvulsant activity. The action included a reduction of the number of seizures, a decrease in clonic and tonic seizures, and a protective effect against mortality, which was conducive to the full survival of the tested animals.

Administration of derivative Les-6291 (100 mg/kg) resulted in a 2.29-fold reduction in the number of seizures per one animal. The duration of the convulsive period was shortened in comparison with the control groups. It is worth adding, that the mortality of animals decreased to 52.38%. After administration of Les-6291 at 53 mg/kg, the number of clonic and tonic seizures were reduced by 50%, 52.38%, respectively. Additionally, the severity of seizures was reduced by 2.23-fold. The compound Les-6291 showed anticonvulsant activity at both doses: 100 mg/kg and 53 mg/kg. The number of animals with clonic seizures was reduced by 100% at a dose of 100 mg/kg and 50% at a dose 53 mg/kg The duration of the seizure period was also shortened to 0.10 ± 0.00 min (at a dose 100 mg/kg) and to 7.08 ± 3.80 min (at a dose 53 mg/kg).

The use of the Les-6290 derivative led to significant reduction of the number of clonic seizures by 16.67%, as well as reduction in tonic seizures by 50% compared to darbufelone. The severity of seizures statistically decreased 1.52-fold and the mortality rate decreased to 69.04% compared to the control group. The compound Les-6296 was found to be the most potent in the experiment and showed high anticonvulsant activity at both doses: 100 mg/kg and 75 mg/kg. The use of Les-6296 at a dose of 100 mg/kg led to a reduction of the number of tonic seizures. It is worth adding, that the administration of a dose of 75 mg/kg reduced the percentage of animals with both tonic (at 69.04%) and clonic (at 16.67%) seizures compared to the control group. In addition, the compound Les-6296, administered at both doses, was responsible for the absolute protective effect against death in the test animals compared to the group of treated with darbufelone.

It is worth noticing, that the presence of the thiazol-2-ylamine substituent in the Position 2 and the 3,5-ditertbutyl-4-hydroxybenzylidene at Position 5 of thiazolidin-4-one cycle are optimal for anticonvulsant activity. These conclusions are based on SAR analysis. In addition, it is worth emphasising, that the best pharmacological effect was obtained for compound Les-6296, which simultaneously became the lead compound. The synthesized derivatives showed a significant level of animals’ protection, and they can be the future-proof compounds for the design of anticonvulsants with optimal pharmacokinetic parameters.

### 2.5. Antidiabetic Activity

Recent scientific research confirms the special role of protein tyrosine phosphatase 1B (PTP1B) inhibitors as potential new antidiabetic drugs [73,74]. The mechanism of action of protein tyrosine phosphatase depends on dephosphorylation of tyrosine residues, which leads to inactivation of proteins responsible for insulin receptor stimulation [75]. As the consequence of said action, PTP1B leads to inactivation of insulin, so it has been suggested that this enzyme is crucial in the development of insulin resistance [76,77,78,79,80,81].

**Figure 32:**
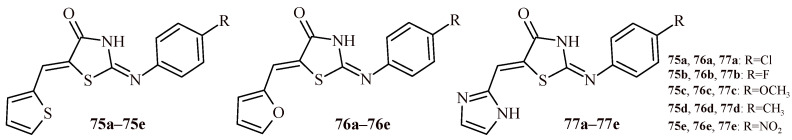
The 5-Heterylmethylidenethiazolidin-4-ones with PTP1B inhibitory activity.

In the published in 2020 study, a number of derivatives with potential antidiabetic activity were synthesized (**75a**–**75e**, **76a**–**76e** and **77a**–**77e**) (Figure 32) [82]. These compounds were tested for their inhibitory activity against PTP1B in vitro using the Calbiochem PTP1B colorimetric kit. Suramin was used as a standard. The inhibitory potency was investigated by measuring the effect of each compound on phosphate production, the results of the inhibition assay are expressed as IC_50_. Generally, the resulting derivatives showed moderate to good inhibition of PTP1B. Their IC_50_ values ranged from 5.88 µM (**76e**) to 29.78 µM (**77a**). For comparison, the result of suramin (the reference substance) was 10.98 µM.

**Figure 33:**
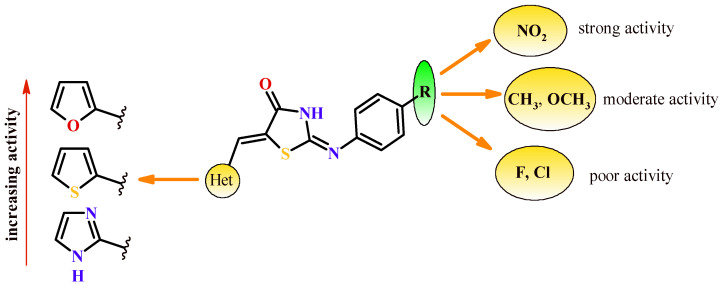
The SAR of different compounds (**75a**–**77e**).

Based on SAR analysis, there is the conclusion that presence of furan ring at Position 5 of thiazolidin-4-one ring is more preferable than two other heterocycles (thiophen and imidazole) for PTP1B inhibitory activity. The compounds (**75e**, **76e** and **77e**) with nitro group in *para* position of phenylamine moiety were most active and showed PTP1B inhibitory activity better than reference drug suramin with IC_50_ values 6.42, 5.88 and 7.93 µM, respectively. The moderate inhibitory effect was exhibited by derivatives (**75c**, **75d**, **76c**, **76d**, **77c** and **77d**) with methyl and methoxy groups in phenylamine moiety. The presence of the electron-withdrawing groups (Cl, F), except Compound **76a**, reduced the activity. The same trends were observed in computational results. The SAR of compounds (**75a**–**77e**) are illustrated in Figure 33.

Jiang et al. synthesized series of 5-(bromobenzylidene)thiazolidine-2,4-dione derivatives and their saturated analogues [83]. The activity of the synthesized derivatives was tested in vitro against recombinant human PTP1B (at a concentration of 20 µg/mL). Initially, the percent of inhibition for all compounds involved in the experiment was measured. Subsequently, their IC_50_ values were determined for best-performing derivatives (>50% inhibition).

**Figure 34:**
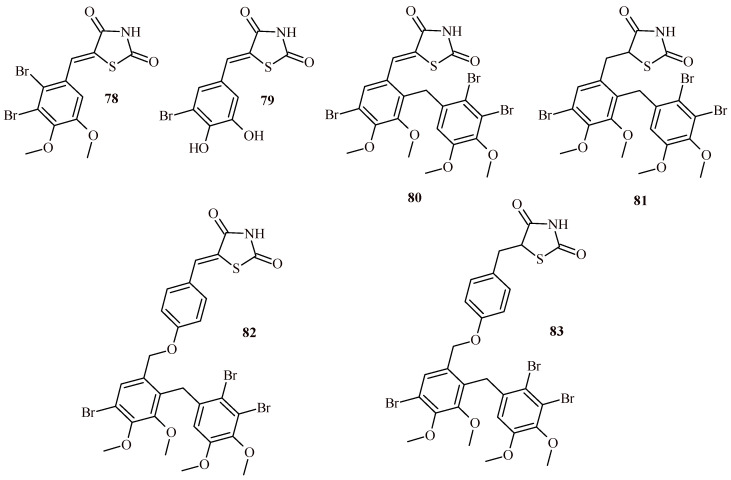
The 5-(Bromobenzylidene)thiazolidine-2,4-dione derivatives (**78**–**80** and **82**) and their saturated analogues (**81**, **83**) as potential protein tyrosine phosphatase 1B inhibitor with antidiabetic properties.

The Derivatives **78** and **79** showed moderate PTP1B inhibition activity with IC_50_ values 8.34 and 10.5 μM, respectively (Figure 34). Introducing the additional 2,3-dibromo-4,5-dimethoxybenzyl group in the bromobenzylidene substituent of Compounds **78**, **79** led to obtaining compounds (**80**, **81** and **83**) with high inhibitory activity against PTP1B with IC_50_ values 0.86, 1.84 and 2.10 μM, respectively. The exception was Compound **82** (IC_50_ = 12.77 μM) that showed moderate effect. The most potent inhibitor PTP1B, Compound **80**, was found to be high selectivity against other PTPs. It is also worth adding, that Compound **80** was proven to be effective in reducing glucose, triglyceride and LDL-C levels in the BKS-db/db mouse model at 50 mg/kg.

Another way to treat diabetes leads to the activity of exocrine enzymes in pancreatic cells. In this case, treatment involves the inhibition of intestinal α-glucosidase and amylase [84,85]. These enzymes are responsible for degradation of starch and oligosaccharides to monosaccharides such as glucose and fructose [85]. Inhibition of these enzymes plays a huge role in antidiabetic activity. Reduction of absorption of simple sugars into the bloodstream would contribute to a reduced fluctuation of sugar levels and would minimize the probability of postprandial hyperglycemia, and this effect is important in pharmacological treatment of diabetes [86]. In a recently published study, a number of thiazolidine-2,4-dione derivatives were synthesized and their influence on the inhibitory activity on the above-mentioned enzymes was investigated.

**Figure 35:**
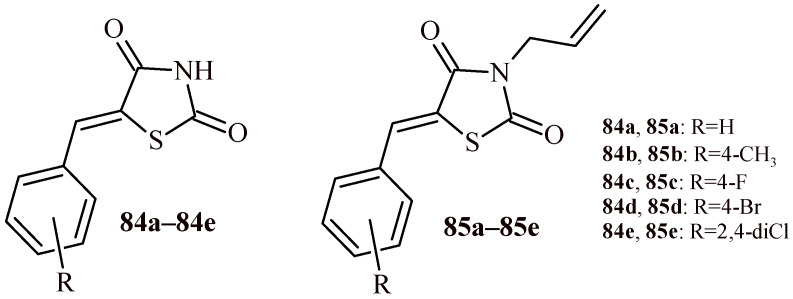
The 5-Arylidenethiazolidine-2,4-diones with α-amylase and α-glucosidase inhibitory activity.

Fettach et al. synthesized series of 5-arylidenethiazolidine-2,4-diones (**84a**–**84e**) and their 3-allyl analogues (**85a**–**85e**) (Figure 35). The synthesized compounds were evaluated for their inhibitory activity towards α-amylase and α-glucosidase enzymes in vitro [87]. It has been shown that all tested derivatives had good or moderate activity toward α-glucosidase (IC_50_ = 43.85–380.10 µM) in comparison with standard inhibitor (acarbose IC_50_ = 97.12 µM). The inhibitory effects of tested compounds against α-amylase were in the range of IC_50_ = 18.19–286.25 µM. Whereas, standard acarbose inhibited α-amylase at IC_50_ value of 2.975 µM.

As the results of research, authors identified lead Compounds **84e**, **85a** and **85e** that showed dual inhibitory effects of α-amylase and α-glucosidase enzymes. The lead Compounds **84e**, **85a** and **85e** inhibited α-amylase with IC_50_: 47.09, 108.14 and 18.19 µM, respectively and α-glucosidase with IC_50_: 84.95, 98.45 and 43.85 µM, respectively. Additionally, the most potent compounds were non-toxic at concentration 2000 mg/kg b.w. in acute toxicity test on mice.

**Figure 36:**
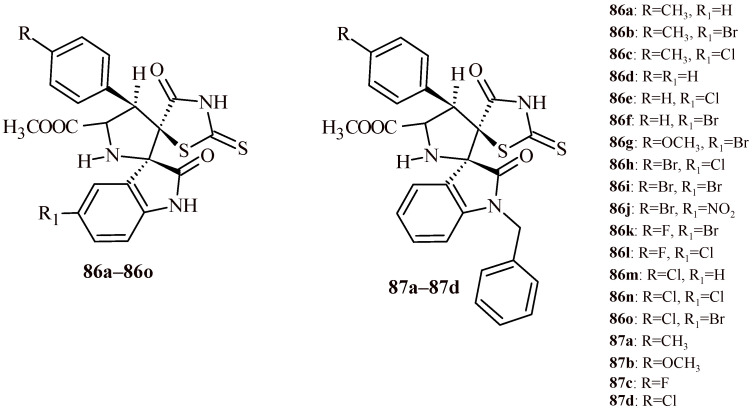
Three cyclic spiro rhodanine derivatives as α-amylase inhibitor.

Toumi et al. [88,89] carried out assay of series of rhodanine derivatives for their α-amylase inhibitory activity. The first series rhodanine-based spirooxindole pyrrolidine derivatives (**86a**–**86o** and **87a**–**87d**) (Figure 36) showed promising activities toward α-amylase enzyme. It is worth noticing that Compounds **86f**, **86i** and **86o** (IC_50_ values 1.49 ± 0.10, 1.50 ± 0.07 and1.57 ± 0.10 µM, respectively) followed by **86j**, **87c** and **87d** (IC_50_ values 1.59 ± 0.08, 1.63 ± 0.09 and1.67 ± 0.09 µM, respectively) exhibited highest inhibitory activity [88]. These results were more potent or comparable to reference drug acarbose (IC_50_ = 1.56 ± 0.07 µM). Other derivatives showed moderate inhibitory effects with IC_50_ values in the range of 1.72–3.06 µM. Moreover, authors carried out in vivo studies to evaluate influence of Compounds **86f**, **86i**, **86j** and **86o** on the blood glucose level in diabetic rats. The daily administration of Compounds **86f**, **86i**, **86j** and **86o** for one month to surviving diabetic rats showed significant decreasing of the serum glucose level by 6.8, 12.3, 5.5 and 9.9%, correspondingly in comparison with untreated diabetic rats.

**Figure 37:**
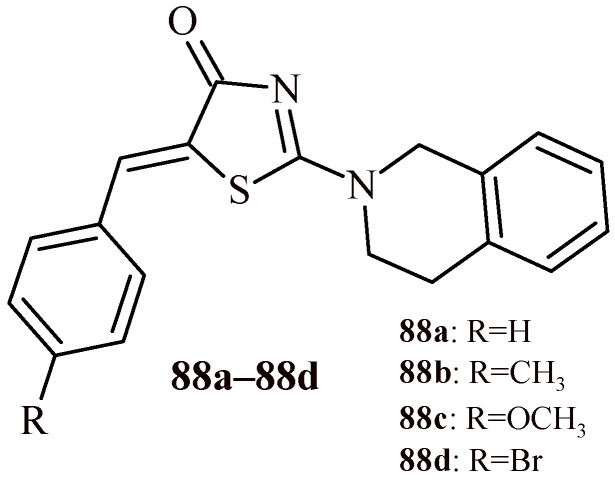
The structures of rhodanine-based tetrahydroisoquinoline derivatives.

The second series of rhodanine-based tetrahydroisoquinoline derivatives (**88a**–**88d**) (Figure 37) exhibited less potential toward α-amylase enzyme in comparison with series 1 (IC_50_ = 5.98–18.06 µM). The most active in vitro Compound **88d** from Series 2 (IC_50_ = 5.98 µM) was tested with alloxan-induced diabetic rats on intestinal α-amylase activity and the blood glucose level. The administration of Compound **88d** at dose of 30 mg/kg daily for 21 days significantly reduced the intestinal α-amylase activity by 21.3% in comparison with untreated diabetic rats, decreased the blood glucose level by 39% [89].

**Figure 38:**
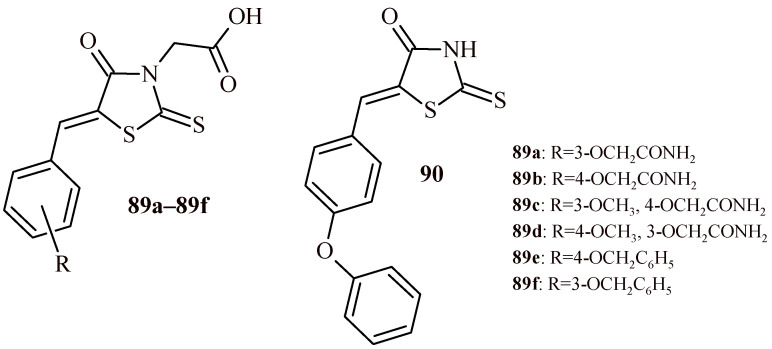
The rhodanine derivatives as α-amylase and α-glucosidase inhibitor.

Szabo et al. evaluated inhibitory activity of rhodanine derivatives (**89a**–**89f** and **90**) towards porcine pancreatic α-amylase (PPA) and α-glucosidase (Figure 38). These rhodanine derivatives previously were reported as inhibitors of human aldose reductase (AR) and PTP1B [90]. Each of the tested compounds (**89a**–**89f** and **90**) showed inhibitory potential against human AR at submicromolar/nanomolar concentration (IC_50_ = 0.052–0.228 µM). On the other hand, only Compounds **89e**, **89f** and **90** showed considerable inhibitory effects towards human PTP1B at concentration <100 µM. The Compounds **89a**–**89f** and **90** possessed diverse inhibitory potential in the tests towards α-amylase and α-glucosidase. Some of them exhibited appreciable effects at concentration ≤100 µM (Compounds **89b**–**89d**), others moderate or insignificant activities (Compounds **89a**, **89e**, **89f** and **90**). As further step of the study, it was evaluated whether inhibitory effects of Compounds **89a**–**89f** and **90** might be due to specific interactions with multiple targets or to non-specific inhibition based on aggregation. Among the tested rhodanines Compounds **89b**, **89e**, **89f** and **90** showed aggregation-based mechanism of action. Whereas Derivatives **89a**, **89c**, **89d** act as multi-target inhibitors without aggregates formation [91].

The series of oxazinyl-thiazolidin-4-ones (**9a**–**9f**), in addition to their antioxidant activity (Figure 4), showed inhibitory activity towards α-amylase and α-glucosidase enzymes in the range of IC_50_ values of 4.08–53.68 µg/mL and 1.01–39.12 µg/mL, respectively [22]. The Compounds **9a** and **9b** revealed inhibition of α-amylase at concentration 9.31 and 4.08 µg/mL. These activities were better than activity of reference drug acarbose (IC_50_ = 11.56 µg/mL). Additionally, these compounds (**9a** and **9b**), as well as Compound **9e,** showed inhibitory effects against α-glucosidase enzyme with IC_50_ values of 1.01, 6.14 and 9.24 µg/mL, respectively. These values also were more beneficial in comparison to acarbose (IC_50_ = 17.23 µg/mL).

**Figure 39:**
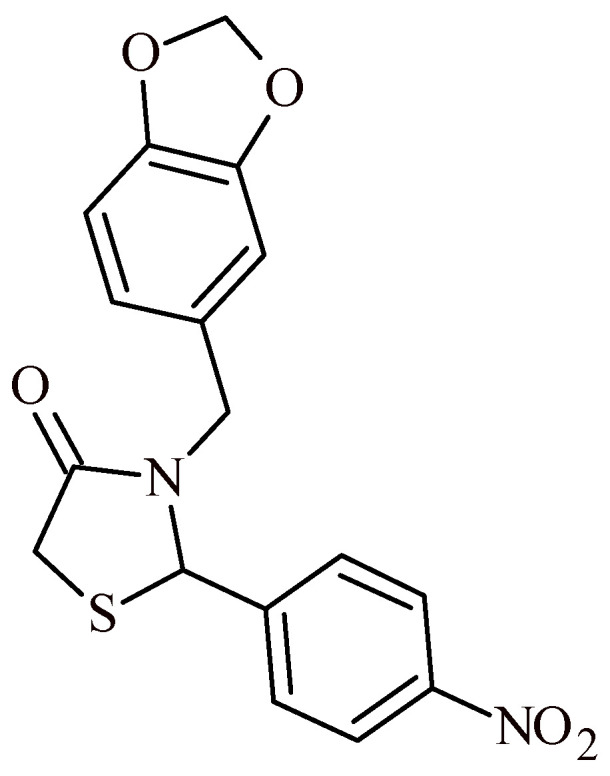
The structure of 3-(benzo[*d*][1,3]dioxol-5-ylmethyl)-2-(4-nitrophenyl)thiazolidine-4-one.

Bilgicli et al. reported, that the piperonyl-based thiazolidine-4-one derivative, namely: 3-(benzo[*d*][1,3]dioxol-5-ylmethyl)-2-(4-nitrophenyl)thiazolidine-4-one, showed very potent inhibition of α-glucosidase at nanomolar concentration (IC_50_ = 5.9 nM) (Figure 39) [92].

### 2.6. Antiparasitic Activity

Leishmaniasis is a mosquito-borne tropical disease, that is directly caused by protozoa of the genus *Leishmania* spp. The infection may take different forms. There are three basic forms of the disease: cutaneous, visceral and mucous [93,94]. Traditionally used drugs face problems such as toxicity, which leads to many side effects, and parasitic resistance [95,96]. Therefore, the need for the synthesis of new compounds that inhibit the parasitic pathways of the *Leishmania* genus is emphasized.

**Figure 40:**
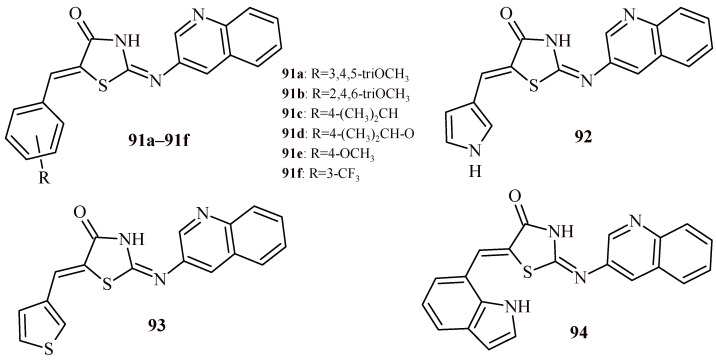
The quinoline-thiazolidin-4-one hybrids as inhibitor of methionine aminopeptidase 1 from *Leishmania donovani*.

Bhat et al. conducted synthesis of quinoline-thiazolidin-4-one hybrids (**91a**–**91f** and **92**–**94**) (Figure 40) and carried out their inhibitory activity towards *Ld*MetAP1 and *Hs*MetAP1 in vitro [97]. Tested hybrids inhibited *Ld*MetAP1 with IC_50_ values in the range of 3.0–123.4 µM and *Hs*MetAP1—54.2–>200 µM. Among the series of quinoline-thiazolidin-4-one hybrids, the best profile was characterized by a derivative with indole moiety (**94**). The Compound **94** (IC_50_ = 3 µM) showed high selectivity towards *Ld*MetAP1 and 20-fold less effectiveness for *Hs*MetAP1 (IC_50_ = 58 µM). It was also found that Compound **94** demonstrated low cytotoxicity in the MTT test using mouse embryonic fibroblast cells (IC_50_ > 150 µM). Moreover, good pharmacokinetics features and good absorption of the drug after oral administration can be predicted because of calculated the LogP and LogS values.

Another important feature of the new derivative is specificity for iron dependent MetAP. MetAP enzymes show high catalytic activity on divalent metals, and they usually show specificity for a specific metal cofactor [98,99,100]. Designing molecules of high sequestration properties of metals can play a significant role towards discovery of new inhibitors. The most important is a high potency drug to the biologically relevant form of the enzyme that is activated by metal. Inhibitors targeting MetAP1, which eliminate *Leishmania* spp. parasites, have the potential to become prime anti-leishmanial drugs. The new inhibitors have the potential to bypass the drug resistance, which is currently experienced in treatment using traditional drugs against leishmaniasis. Discovery of drugs similar to a synthesized derivative, which shows selectivity to *Ld*MetAP1, may help with development of new chemotherapeutic molecules.

**Figure 41:**
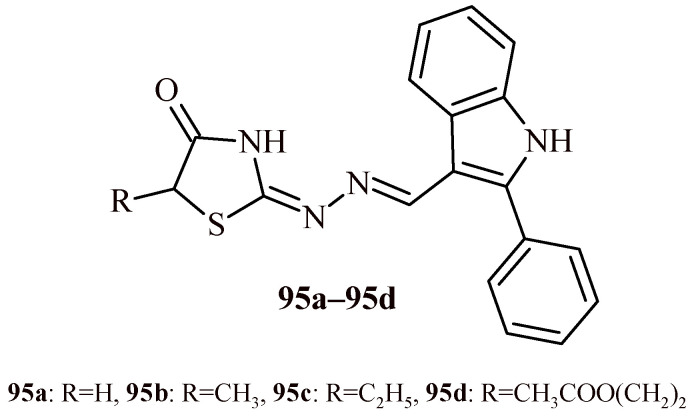
Thiazolidin-4-one-indole hybrids with antitrypanosomal and antileishmanial activities.

Schadich et al. carried out the evaluation of 29 compounds belong with different thiazolidine-based hybrids against *Leishmania major* FV1 strain. Some of the tested compounds showed antitrypanosomal activity against *Trypanosoma brucei* and *Trypanosoma gambiense* [101]. The most effective was group of thiazolidin-4-one-indole hybrids (**95a**–**95d**) (Figure 41). The Compounds **95a**–**95d** showed good antileishmanial activity against *L. major* in the range of IC_50_ values of 1.01–3.51 µM, that was better than reference drug miltesofine (IC_50_ = 8.18 µM). The Hybrids **95a**–**95d** also revealed good antitrypanosomal activity against *T. gambiense* (IC_50_ = 0.11–0.23 µM). Additionally, Compounds **95c** and **95d** exhibited strong activity against *T. brucei* with IC_50_ 0.03 and 0.06 µM, respectively. The most active compounds—**95a**–**95c** showed low cytotoxicity to normal human fibroblasts (IC_50_ > 50 µM) and good therapeutic index (>29). Based on SAR analysis, it can be said, that the advantageous for antileeishmanial and antitrypanosomal activities is the unsubstituted Position 3 in the thiazolidine ring and the lack of a bulky substituent in the five positions of this ring.

Another method of inhibiting of the development of *Leishmania* spp. parasites is the inhibition of the biochemical pathway of the formation of folates. The *Leishmania* spp parasites receive tetrahydrofolate by action of the enzyme dihydrofolate reductase—thymidylate synthetase. No effective drugs that act on this subunit such as methotrexate follow directly from the presence of pteridine reductase (PTR1) [102]. The conducted tests showed unequivocally, that PTR1 is an enzyme necessary for the growth and development of *Leishmania* spp. parasites [103]. Moreover, the selective inhibition of PTR1 is sufficient to inhibit the growth of the parasite, and the new drugs against leishmaniasis based on blocking this enzyme can be very effective [104,105].

**Figure 42:**
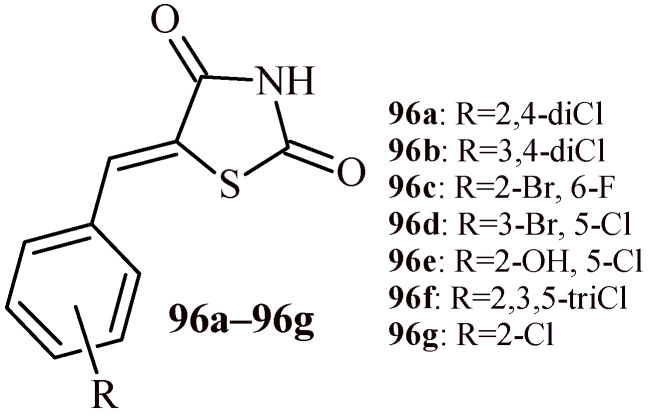
The 5-(Halogenbenzylidene)thiazolidine-2,4-dione derivatives with antileishmanial activity.

A series of thiazolidine-2,4-dione derivatives (**96a**–**96g**) with antileishmanial activity was synthesized by Neri et al. (Figure 42) [106]. Their activity is based on the above presented mechanism. The Compounds **96a**–**96g** showed antileishmanial activity at EC_50_ concentration range of 44.16–70.98 µM (for *Leishmania braziliensis*) and 23.45–68.77 µM (for *Leishmania infantum*). The most effective compounds against *L. infantum* (**96b**, **96d** and **96f**, EC_50_ = 23.45, 35.90 and 30.36 µM, respectively) had halogen substituent in Positions 3, 4 or 5 of phenyl group. The introduction of halogen in Positions 2 or 6 led to decreased activity. The main insufficiency of these compounds is their cytotoxicity. Tested compounds showed LD_50_ values in the range of 26.67–61.29 µM against WI-26VA4 cell line, except Compound **96c** (LD_50_ > 100 µM). Only two compounds (**96b** and **96c**) exhibited a selectivity index more than one.

The inhibition constant (Kd) of *Lm*PTR1 was determined by ThermoFluor^®^ assays. According to this method, Compound **96f** (Kd = 18.56 μM) has the lowest Kd value, followed by Compound **96b** (23.12 μM), **96d** (25.35 μM), **96e** (89.98 μM), and **96g** (98.11 μM). Compound **96f** seems to have higher affinity to *Lm*PTR1 than previous lead compound (**96d**). The conducted analyzes show, that the presence of small lipophilic substituents at the *meta* and/or *para* positions are necessary for the efficacy of the compounds against *Lm*PTR1. *Ortho* positioned lipophilic substituents affect the biological activity of the compound against *L. infantum*. Moreover, the derivative having in its structure chlorine atoms in Positions 3 and 4 in phenyl group (compound **96b**) was characterized by the best profile of activity from the group of synthesized compounds with the greatest activity. It was considered the most interesting derivative due to its enzymatic and cellular properties.

**Figure 43:**
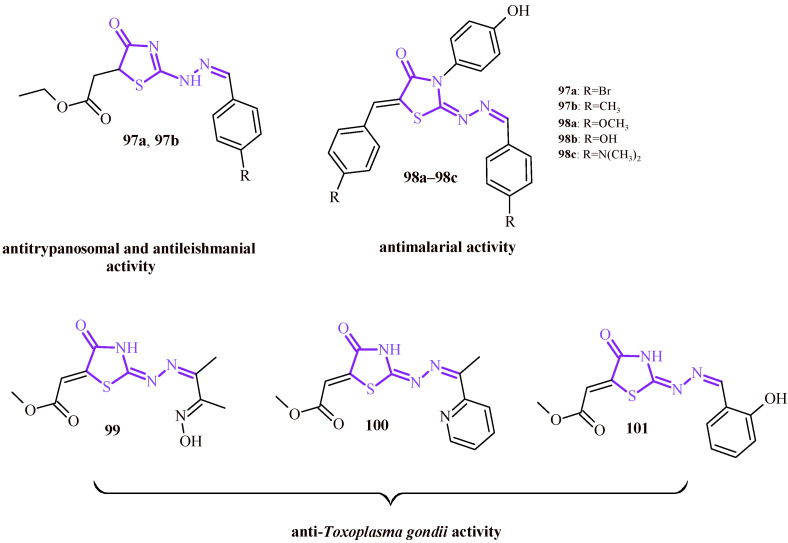
The 2-ylidenehydrazinylidenethiazolidin-4-one derivatives with antiparasitic activities.

It is also worth emphasizing the importance of the 2-ylidenehydrazinylidenethiazolidin-4-one moiety for antiparasitic activity (violet color in Figure 43). The (4-oxothiazolidin-5-yl)acetic acid Derivatives **97a** and **97b** showed good antitrypanosomal activity against trypomastigote form with EC_50_ values of 8.45 and 29.26 µM, respectively. These values were better than those for standard benzimidazole (EC_50_ = 34.5 µM). Furthermore, the Compounds **97a** and **97b** demonstrated better IC_50_ values for both *Leishmania amazonensis* (IC_50_ = 14.63 and 13.35 µM) and *Leishmania infantum* (IC_50_ = 14.63 and 13.35 µM) in comparison with reference drug miltefosine (36.31 and 53.71 µM) [107].

Kryshchyshyn et al. reported that 5-arylidene-3-(4-hydroxyphenyl)thiazolidin-4-ones (**98a**–**98c**) showed antimalarial activity against *Plasmodium falciparum*. The IC_50_ values were in the range of 2.32–3.50 µM [108].

Another study concerns to anti-*T. gondii* activity. The group of (4-oxothiazolidin-5-ylidene)acetic acid derivatives (**99**–**101**) showed activity against *T. gondii* with IC_50_ values of 0.46, 0.20 and 0.66 µM, respectively. In addition, besides the anti-*T. gondii* activity, Compounds **99**–**101** had also lower toxicity towards the host cells (TD_50_ = 60, 206, 125 µM, correspondingly) [109].

**Figure 44:**
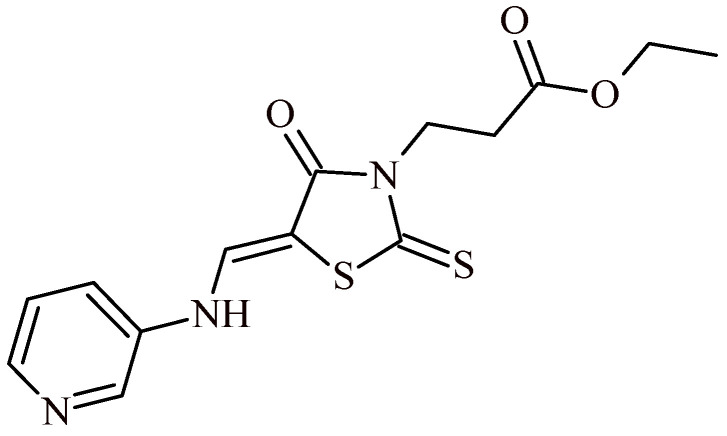
The structure of ethyl 3-[4-oxo-5-[(3-pyridylamino)methylene]-2-thioxothiazolidin-3-yl]propanoate (**102**).

The rhodanine-3-carboxylic acid Derivative **102** (Figure 44) exhibited also antitrypanosomal effects against *T. brucei brucei* and *T. brucei gambiense* at half maximal inhibitory concentration 19.19 and 5.03 µM. Moreover, Compound **102** showed low cytotoxicity towards fibroblast cells (CC_50_ = 182.41 µM) and good selectivity (SI = 9.5 and 36.2, respectively) [110].

The good antitrypanosomal activity against *T. brucei brucei* showed two thiazolidin-4-one-diclofenac hybrids, namely: [2-(2,6-dichlophenylamino)phenyl]acetic acid *N*-(3-ethyl-4-oxo-2-thioxothiazolidin-5-ylidenemethyl)hydrazide and [2-(2,6-dichlophenylamino)phenyl]acetic acid *N*-(2,4-dioxothiazolidin-5-ylidenemethyl)hydrazide. Their IC_50_ values were 7.06 and 4.8 µM, correspondingly [68].

### 2.7. Antimicrobial Activity

Despite the wide number of groups of compounds used in microbial and fungal infections, diseases continue to grow in strength and their treatment is a serious problem of a medical nature. Widespread and extensive use of antibiotics contributed to the development of pathogen resistance resulted the need to synthesize new antimicrobial drugs with a mechanism of action bypassing antibiotic resistance. The emergence of multi-resistant pathogenic strains is the undoubted challenge of modern medicine, and the prevention of infectious diseases is a serious global problem [111,112].

**Figure 45:**
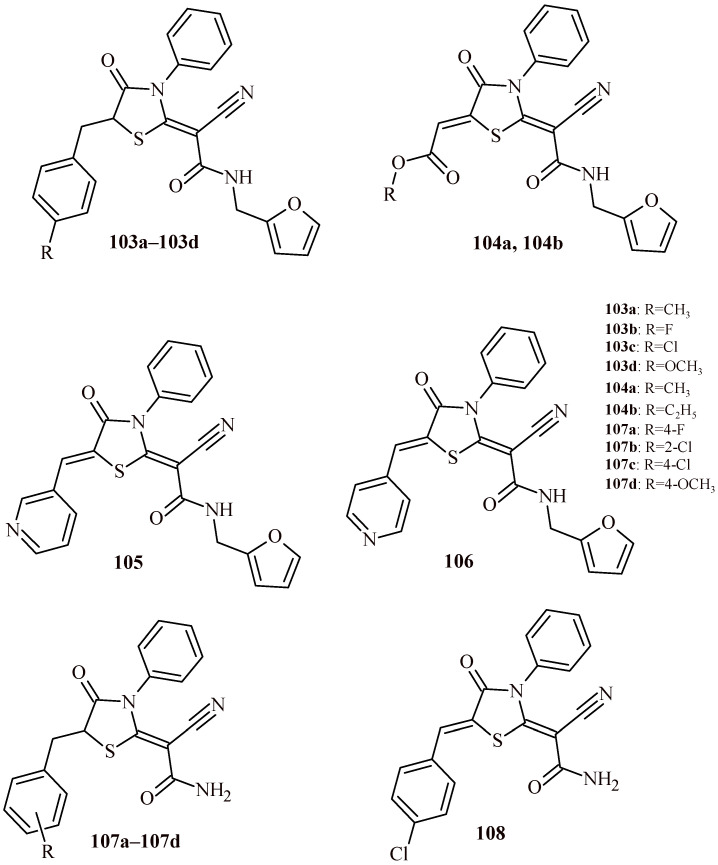
The structures of 4-oxothiazolidin-2-ylidene derivatives with potential antimicrobial activity.

Chaban et al. performed synthesis and antimicrobial screening of 4-oxothiazolidin-2-ylidene derivatives [113]. In the first stage of antimicrobial screening, it was evaluated the growth inhibition (%) of tested compounds against methicillin-resistant *Staphylococcus aureus* (ATCC 43300) (Gram-positive) and four Gram-negative strains: *Escherichia coli* (ATCC 25922), *Klebsiella pneumoniae* (ATCC 700603), *Acinetobacter baumannii* (ATCC 19606), and *Pseudomonas aeruginosa* (ATCC 27853) as well as two fungal strains: *Candida albicans* (ATCC 90028) and *Cryptococcus neoformans* var. *Grubii* (H99; ATCC 208821). The compounds (**103a**–**103d**, **104a**, **104b**, **105** and **106**) (Figure 45) were selected for the next stage of antimicrobial screening and showed significant microbial growth inhibition. The minimal inhibitory concentration (MIC) measurements were performed. The ceftriaxone and amphotericin were used as reference drugs for antibacterial and antifungal activity, respectively. The Compounds **103b** and **103c** showed antibacterial activity (MIC = 4–32 µg/mL) against all tested bacterial strains comparable to ceftriaxone. The highest antifungal effect was exhibited by Compounds **104a** and **104b** that inhibited growth of *C. neoformans* with MIC values 4 and 8 µg/mL, respectively. Worth noticing, except **105** and **106** (CC_50_ = 8 and 6.86 µg/mL, respectively) that compounds (**103a**–**103d**, **104a** and **104b**) revealed low cytotoxicity against human embryonic kidney cells HEK-293 (CC_50_ > 32 µg/mL). The Compounds **107a**–**107d** and **108** with unsubstituted amido group showed high antibacterial activity against *S. aureus* with growth inhibition in the range of 85.3–97.9% [114].

In the paper published by Kumar et al. [20], antimicrobial activity of 5-arylidenethiazolidine-2,4-dione derivatives (**5a**–**5s** and **6**) was discussed (Figure 2). Synthesized TZD derivatives were evaluated for their in vitro antimicrobial activity by serial tube dilution procedure. The antibacterial screening results were found to be comparable with cefadroxil, a standard drug used in bacterial infections and exceeded fluconazole in terms of antifungal activity. The screening revealed that Compounds **5l** and **5d** were moderately active against *S. aureus* (MIC of 17.9 µM and 18.2 µM, respectively). Compounds **5o** and **5i** were moderately active against *Bacillus subtilis* (MIC of 18.5 µM and 18.6 µM, respectively), Compounds **5l** and **5h** were effective against *K. pneumoniae* (MIC = 17.9 and 18.6 µM, respectively). Compound **5n** (MIC = 18.5 µM) and Compound **5h** (MIC = 18.6 µM) exhibited promising activity against *Salmonella typhi*. The antifungal screening results revealed that the Compounds **5l** (MIC = 17.9 µM) and **5n** (MIC = 18.5 µM) had good activity against *C. albicans* and *Aspergillus niger*. The most active derivative against *C. albicans* was **5k** (MIC = 16.1 µM).

**Figure 46:**
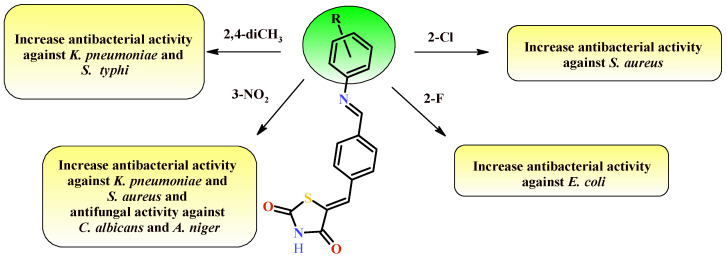
Influence of substituents on antimicrobial activity compounds (**5a**–**5s**).

Firstly, substitution of electron-donating methyl group at *ortho* and *para* position in Compound **5h** increased the antibacterial potential against *S. typhi* and *K. pneumoniae*. Presence of an electron-withdrawing nitro group (**5l**) in *meta* position enhanced antibacterial potential against *K. pneumoniae* and *S. aureus* as well as antifungal activity against *C. albicans* and *A. niger*. Furthermore, presence of electron-withdrawing fluorine substituent at *ortho* position of the synthesized Compound **5j** enlarged the antibacterial potential against *E. coli* (Figure 46). No doubt, that thiazolidin-4-one derivatives are an appropriate base for the synthesis of subsequent derivatives with antimicrobial activity.

**Figure 47:**
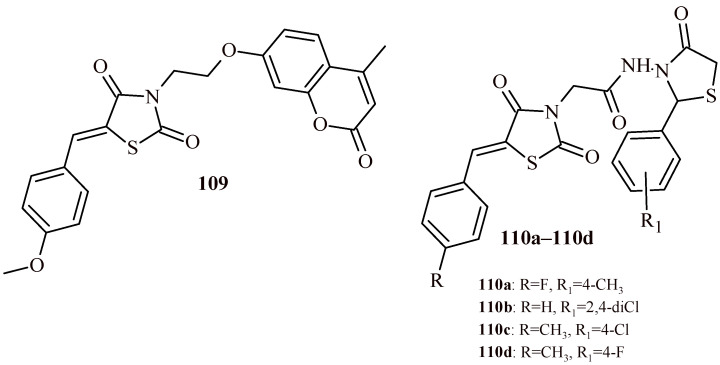
TZD derivatives with antimicrobial potential.

It is worth to pay attention to Compound **109** (Figure 47) among the TZD derivatives, that exhibited good antibacterial as well as antifungal activity. The minimal inhibitory concentration against *S. aureus* and *B. subtilis* was 0.5 µg/mL. This derivative was active against Gram-negative strain *E. coli* and *P. aeruginosa* at MIC = 1 µg/mL. The Compound **109** showed antifungal activity also at Concentration 1 µg/mL against *Aspergillus flavus*, *Trichoderma harzianum*, *Penicillium chrysogenum* and *C. albicans*. Al these values were better than for reference drugs ciprofloxacin (MIC = 2 µg/mL) and fluconazole (MIC = 2 µg/mL) [115].

The group of Compounds **110a**–**110d** showed antibacterial activity against Gram-positive (*S. aureus*, *Bacillus cereus*, *Micrococcus luteus*) and Gram-negative (*Pseudomonas fluorescens*, *E. coli*, *Flavobacterium devorans*) strains with MIC values in the range of 2–4 µM. These results were comparable with reference drugs ampicillin, kanamycin and chloramphenicol [116].

Among the thiazolidin-4-ones (**111a**, **111b**) with trifluoromethoxy group, Compound **111a** showed antibacterial activity at minimal inhibitory concentration of 100 µg/mL against *S. aureus* and *Listeria monocytogenes* strains. Slightly better activity was exhibited by their chlorosubstituted analogues (**111c**, **111d**) against *L. monocytogenes* and *P. aeruginosa* resistant strain with MIC = 60 µg/mL [117,118].

**Figure 48:**
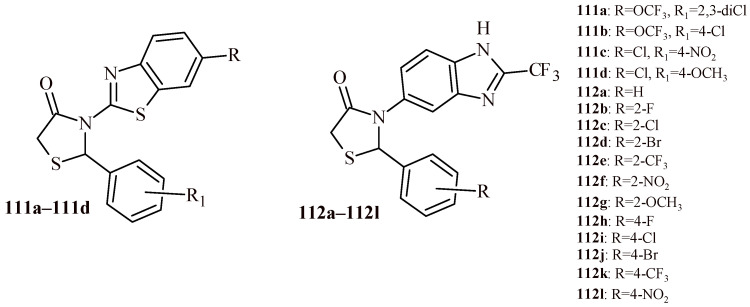
The structures of thiazolidin-4-ones with benzothiazole and benzimidazole moieties.

The replacement of benzothiazole moiety by the benzimidazole fragment (Compounds **112a**–**112l**) led to increasing antibacterial activity both for Gram-positive and Gram-negative strains (Figure 48). The most active compound against S. aureus was Derivative **112k** with trifluoromethyl substituent (MBC = 0.12 µM). The compounds with trifluoromethyl and bromo groups (**112d**, **112e** and **112j**) were most active against *K. pneumoniae* (MBC = 0.14 µM). Whereas Compound **112l** showed activity against three Gram-negative strains (*Salmonella thyphimurium*, *K. pneumonia* and *E. coli*) with MBC value 0.15 µM [119].

**Figure 49:**
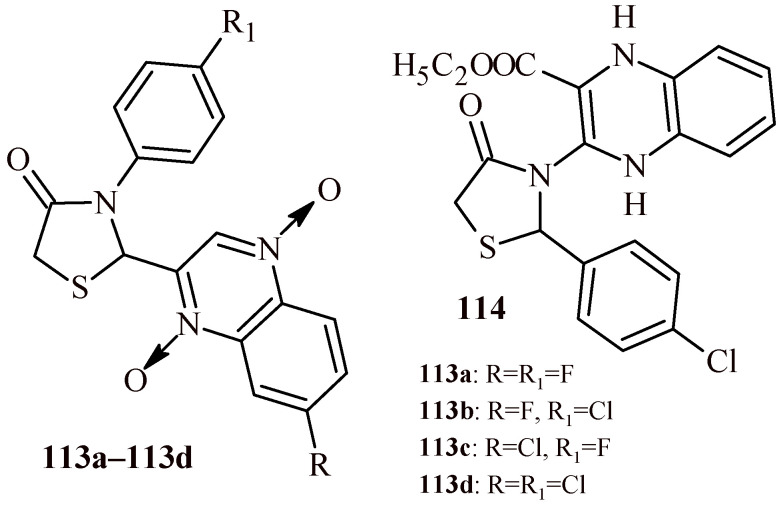
The structures of thiazolidin-4-ones with quinoxaline moiety.

Zhang et al. reported antifungal activity of Compounds **113a**–**113d** against *C. albicans*, *Candida tropicalis*, *Aspergillus fumigatus* and *C. neoformans* fungal strains with MIC in the range of 2–4 µg/mL [120]. Other derivative with quinoxaline moiety (**114**) showed comparable antibacterial and antifungal activity to reference drugs ampicillin (inhibition zone 23–28 mm) and amphotericin B (inhibition zone 14–20 mm) (Figure 49). The inhibition zone of **114** against *S. aureus* and *E. coli* were 24 and 19 mm, respectively. The Compound **114** inhibited growth of *A. flavus* and *C. albicans* with inhibition zone 13 mm [121].

The Compounds **11** and **43a** (Figure 7 and Figure 18) exhibited strong antimicrobial activity against four bacterial (*E. coli*, *K. pneumoniae*, *S. aureus* and *B. subtilis*) and three fungal (*A. niger*, *Aspergillus oryzae* and *C. albicans*) strains with MIC = 1.5 µg/mL. This activity was comparable or slightly better than reference drugs streptomycin, ciprofloxacin and fluconazole [25,26].

**Figure 50:**
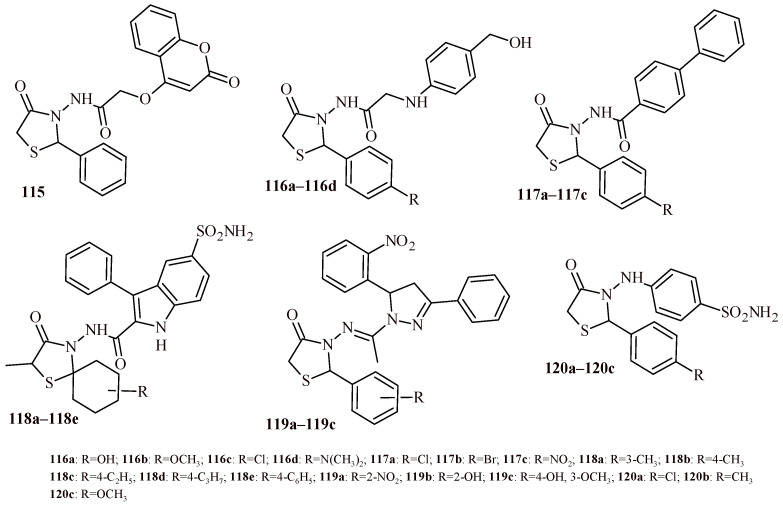
The structures of 3-amino-2-aryl/alkylthiazolidin-ones with antimicrobial activity.

Among the 3-amino-2-aryl/alkylthiazolidin-one derivatives, Compound **115** (Figure 50) showed higher inhibitory activity (inhibition zone—8–17 mm) than standard chloramphenicol (5–15 mm) to concentration 150 µg/mL against all tested strains (*Proteus vulgaris*, *E. coli*, *K. pneumoniae*, *P. aeruginosa* and *S. aureus*) [122]. Among the series of **116a**–**116d**, Compound **116c** with chloro substituent showed the antibacterial activity against *E. coli* practically equivalent to trimethoprim and better than trimethoprim against *Acinetobacter baumannii* and *Streptococcus pyogenes* [123]. The biphenylbased thiazolidine-4-ones (**117a**–**117c**) showed good antimicrobial against bacterial (*E. coli*, *S. aureus*, *B. subtilis*) and fungal (*A. niger*, *C. albicans*) strains, except **117c** that exhibited moderate effect against fungal strains [124].

The spiro derivatives (**118a**–**118e**) showed good to moderate antibacterial activity against *S. aureus* ATCC 29213 MSSA and *S. aureus* ATCC 33951 MRSA strains. The Compounds **118c** and **118d** were most active against *S. aureus* MSSA strain at MIC = 9.765 µg/mL. The Compound **118b** inhibited growth of *S. aureus* MSSA, while Compound **118a** was most active against *S. aureus* MRSA at concentration 19.53 µg/mL. The moderate antibacterial effect showed Compounds **118b** (*S. aureus* MRSA), **118e** (*S. aureus* MSSA) with MIC = 39.06 µg/mL and **118c**, **118d**, **118e** (*S. aureus* MRSA) as well as **118a** (*S. aureus* MSSA) at concentration 78.12 µg/mL. The cyclopentane spiro derivatives, analogues of **118** were no active (MIC > 2500 µg/mL) [125].

Among the thiazolidine-4-one derivatives with pyrazole moiety, the most active were Compounds **119a**–**119c**, that showed antibacterial activity at MIC = 12.5 µg/mL against *B. subtilis* (**119a**), *S. aureus* (**119c**) and *P. aeruginosa* (**119b**) [126]. Unfortunately, none of the tested Compounds **120a**–**120c** was active against bacterial and fungal strains at concentration less tha 50 µg/mL [127].

**Figure 51:**
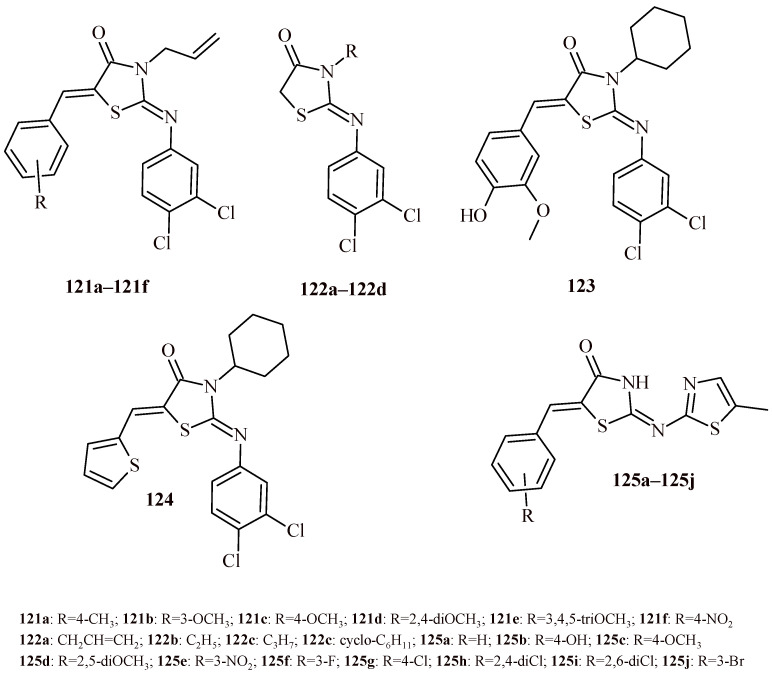
The structures of 2-iminothiazolidin-4-one derivatives.

Hammad et al. carried out evaluation of 3-allylthiazolidin-4-ones for their antimicrobial potential. The Compounds **121a**–**121e** with methyl and methoxy groups and **121f** with *para*-nitro group (Figure 51) exhibited slight antibacterial activity against Gram-positive and Gram-negative bacterial strains with percent of growth inhibition 16–24% at aconcentration of 32 µg/mL. Additionally, Compound **121f** inhibited *S. aureus* HG001 biofilm formation with percentages of 35% and 54% at a concentration of 32 and 64 µg/mL, respectively. Unsubstituted compound in Position 5 of thiazolidine ring (**122a**) showed full growth of inhibition (100%) against *C. neoformans* at concentration at 32 µg/mL [128].

Continuing their research, Hammad et al. carried out the evaluation of other 3-alkylthiazolidin-4-ones (**122a**–**122d**, **123** and **124**) for their antibacterial activity. Among the unsubstituted derivatives in Position 5 (**122a**–**122d**), three compounds (**122a**–**122c**) showed antibacterial effect against *E. coli* JW55031 (tolC Mutant) at concentration at 16 µg/mL. Compounds **123** and **124** were active against *S. aureus* MRSA with MIC 32 and 64 µg/mL, correspondingly. Moreover, Compound **123** showed activity against clinically important Gram-positive bacterial pathogens (*S. aureus*, *Enterococcus faecalis*, *Enterococcus faecium*, *L. monocytogenes*, *Streptococcus pneumoniae*). Compound **123** inhibited growth of tested pathogens at concentration in the range of 8–128 µg/mL, and *L. monocytogenes* was the most sensitive clinical isolate to **123** (MIC = 8 µg/mL). Additionally, Compound **123** at 2 x MIC concentration, inhibited about 17% of *S. aureus* MRSA USA 300 biofilm mass [129].

The thiazolidin-4-one-thiazole hybrids (**125a**–**125j**) were tested for their antibacterial, antifungal as well as antibiofilm activities. All compounds showed antibacterial effects, but their potency was different (MIC = 26.3–378.5 µM). Compound **125e** with nitro group demonstrated the highest antibacterial activity with MIC in the range of 43.3–86.7 µM. This compound is six-fold more potent than ampicillin and three-fold than streptomycin against all eight bacterial strains. All compounds showed very good antifungal activity against eight fungal strains at MIC of 27.7–578 µM. Most active was Compound **125g** with MIC = 59.6–119.2 µM and MFC = 119.2–238.4 µM. Furthermore, Compounds **125f**, **125i** and **125j** reduced the biofilm-forming abilities of *P. aeruginosa*. The percentage of reduction by these compounds was above 50% at concentration equal to their MIC [130].

**Figure 52:**
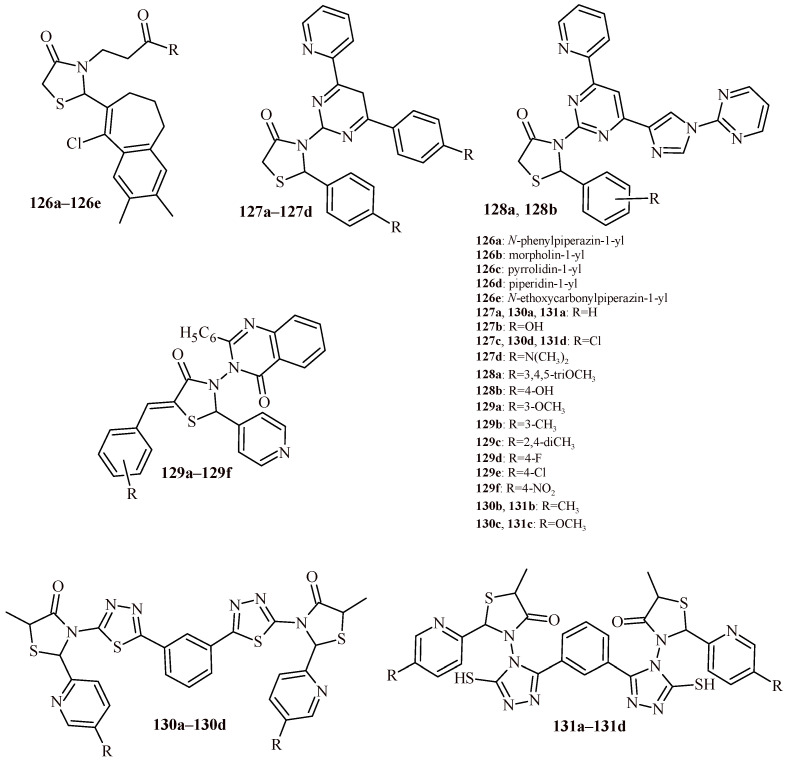
The structures of thiazolidin-4-one derivatives with various heterocyclic substituents in Position 3.

The series of thiazolidine-4-one-benzosuberone derivatives (**126a**–**126e**) was tested for their antimicrobial effects (Figure 52). Compounds **126c** and **126d** with pyrrolidine and piperidine moieties were the most active against bacterial (*M. luteus*, *S. aureus* MTCC 96, *S. aureus* MLS-16 MTTC 2940, *B. subtilis*, *E. coli*, *Klebsiella planticola*, *P. aeruginosa*), as well as fungal (*C. albicans*) strains. MIC were in the range of 1.9–7.8 µg/mL. Compound **126c** showed highest activity against *B. subtilis* MTCC 121 at concentration 1.9 µg/mL. Additionally, Compounds **126c** and **126d** were two-fold more active than miconazole (3.9 vs. 7.8 µg/mL) against *C. albicans* [131].

Compounds **127a**–**127d** generally showed moderate antibacterial and antifungal activities, except Compound **127c**. This derivative demonstrated comparable antifungal activity (inhibition zone—14 and 21 mm) with amphoterecin B (inhibition zone—15 and 19 mm) towards *A. niger* and *C. albicans* strains, respectively [132]. Other thiazolidine-4-one derivatives, with pyrimidine moiety (**128a**, **128b**) demonstrated good antibacterial activity with MIC in the range of 3.12–12.5 µg/mL against *S. aureus*, *S. epidermidis*, *E. coli* and *Proteus mirabilis*. These values were comparable to standard ciprofloxacin. The most sensitive to Compounds **128a**, **128b** was *S. epidermidis* at concentration 3.12 µg/mL [133].

According to Desai et al., thiazolidin-4-one quinazoline hybrids (**129a**–**129f**) exhibited good to moderate antibacterial effect. Compounds **129a** and **129b**, with methoxy or methyl group were most active against *S. aureus* and *E. coli*, correspondingly at concentration 12.5 µg/mL. These values were better, than for standard chloramphenicol or ciprofloxacin (50 µg/mL). The Derivatives **129d**–**129f** with *para*-fluoro, *para*-chloro and *para*-nitro showed comparable antifungal activity to nystatin and griseofulvin (MIC = 100–250 µg/mL) against *C. albicans* and *A. niger* [134].

The screening of symmentrical bis-thiazolidin-4-ones (**130a**–**130d** and **131a**–**131d**) for their antimicrobial activity showed good antifungal activity and moderate antibacterial effects in some cases. Therefore, Compounds **130c** and **131d** showed comparable zone of inhibition to ketoconazole (19 mm vs. 18mm) against *A. flavus*, while **130a**, **131a** and **131d** demonstrated comparable activity to ketoconazole against *C. albicans* (19–22 mm vs. 24 mm) [135].

**Figure 53:**
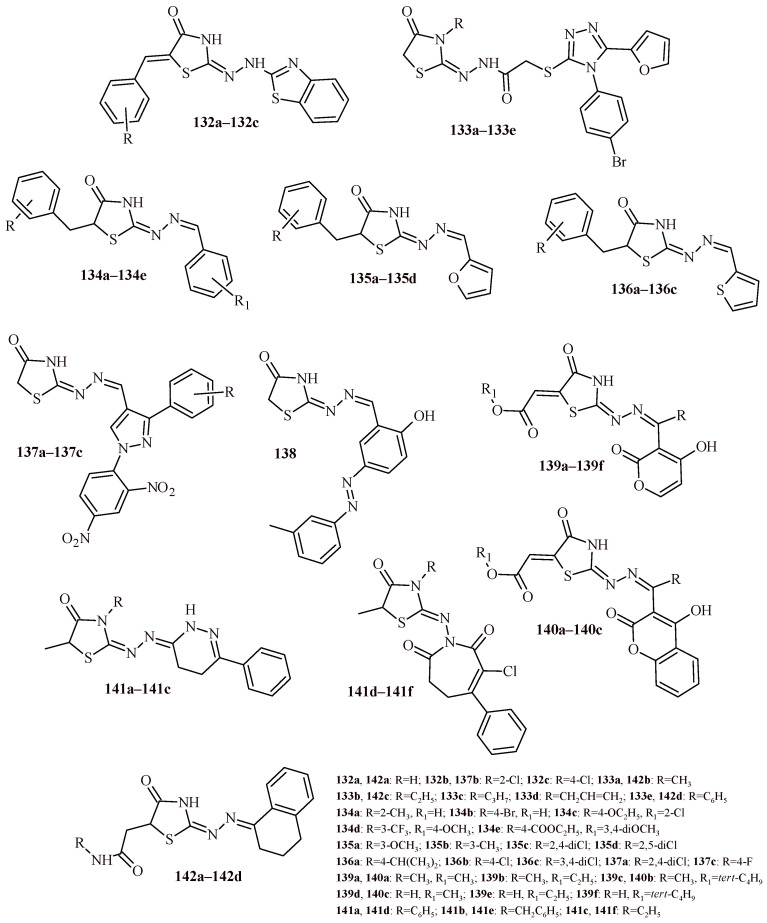
The structures of 2-hydrazynilidenethiazolidin-4-ones as potential antimicrobial agents.

The Compounds **132a**–**132c** (Figure 53) and their inclusion complex with β-cyclodextrin were tested on antibacterial activity against *E. coli*, *S. aureus* and *P. vulgaris*. The results showed that inclusion complex with Compounds **132a**–**132c** had higher activity than simple small molecules (**132a**–**132c**). For example, inhibition zones of Compound **132c** were in the range of 15–16 mm, while it inclusion complex showed inhibition zone at 19–21 mm [136].

Compounds **133a**–**133e** demonstrated moderate to weak antibacterial activity against *E. coli*, *S. aureus* and *P. aeruginosa* at concentration of 32–>64 µg/mL [137].

Chaban et al. reported generally weak antimicrobial activity of Compounds **134a**–**136c**. Their percent of growth inhibition (GI) was less than 50%, except Compounds **135d** and **136c**, that showed GI = 72.4 and 64.4 % against S. aureus [138,139].

However, Compounds **137a**–**137c** showed high antibacterial activity against *S. aureus* MRSA and *S. aureus* ATCC 25923. The chloro and dichloro Derivatives **137b** and **137a** were most potent against both *Staphylococcus* strains with MBC of 0.16 µM and 1.3 µM, respectively. Compound **137c** also was high active against MRSA (MBC = 1.3 µM) [140].

The diazo Derivative **138**, as the only one in the series, exhibited moderate antibacterial effect against *B. subtilis* (inhibition zone = 13 mm), *S. aureus* (9 mm) and *E. coli* (10 mm) [141].

The thiazolidine-4-one with 2*H*-piran moiety **139f**, **140a**–**140c** demonstrated improvement of antibacterial activity against *B. subtilis* with zone of inhibition 15–23 mm as well as **139d**, **139e** and **139f** against *M. luteus* (16–21 mm). These values of inhibition zone were better than the same parameter for reference drugs erythromycin and tetracycline [142].

The Compounds **141a**–**141f** showed comparable antibacterial and antifungal activity to reference drugs cefotaxime-sodium and nystatin. The **141a**, **141b** and **141e** were most effective against *K. pneumoniae* with MIC = 1 µmol/mL. The **141e** was also active against *C. albicans* at concentration 1 µmol/mL [143].

Compounds **142a** and **142b** exhibited better antibacterial activity than reference drugs gentamycin and amoxicillin against *S. aureus* ATCC 43300 methicillin-sensitive strain. The inhibition zone of **142a** was 18 mm vs. 12 and 15 mm for gentamycin and amoxicillin, respectively. Compound **142b** showed bactericidal effect. The Compound **142d** demonstrated highest activity against *C. albicans* (fungicidal effect) and it was more active than nystatin used as standard. Derivative **142c** was inactive against both above mentioned strains [144].

**Figure 54:**
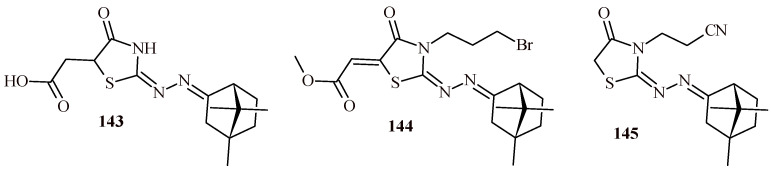
The structures of camphor-thiazolidine-4-one hybrids.

As reported Abo-Bakr et al. camphor-thiazolidin-4-one hybrids (**143**–**145**) (Figure 54) showed antibacterial activity against *B. subtilis* with inhibition % at 65.2, 73.9 and 60.9, respectively. Moreover Compound **144** exhibited 50% of inhibition against *E. coli*. Additionally, Derivatives **143** and **144** were active against *C. albicans* (inhibition at 51.8 and 66.7%) and *A. flavus* (inhibition at 76 and 84%) [145].

Konechnyi et al. also reported the moderate effects of 3-[5-(1*H*-indol-3-ylmethylene)-4-oxo-2- thioxothiazolidin-3-yl]-propanoic acid against *C. albicans* (MIC = 25 µg/mL) and 2-[5-(1*H*-indol-3-ylmethylene)-4-oxo-2-thioxothiazolidin-3-yl]-ethanesulfonic acid against *E. coli* and *S. aureus* (MIC = 25 µg/mL) [146].

### 2.8. Antitubercular Activity

**Figure 55:**
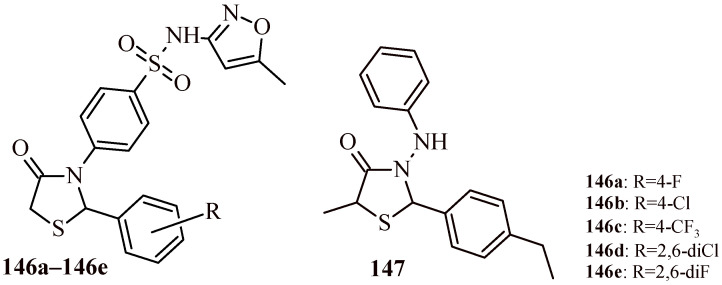
The 2-Arylthiazolidin-4-ones with antimycobacterial activity.

The thiazolidine-4-one derivatives show also antitubercular activity. Thus, a series of sulfamethaoxazole-thiazolidin-4-one hybrids (**146a**–**146e**) (Figure 55) showed excellent antitubercular effects against *Mycobacterium bovis* BCG and *Mycobacterium tuberculosis* H37Ra strains with IC_90_ values in the range of 0.058–0.22 and 0.43–5.31 µg/mL, respectively [147]. All most active compounds contain halogen substituent in 2-phenyl group at *ortho* or *para* positions.

A series of 2-aryl-5-methylthiazolidin-4-ones was synthesized by Ekinci et al. All compounds of this series were evaluated for antimycobacterial activity in vitro against *M. tuberculosis* H37Rv strain by microplate alamar blue assay (MABA) [148]. Only Compound **147** (Figure 55) with 2-(4-ethylphenyl) substituent showed moderate antimycobacterial effect with MIC = 12.5 µg/mL. Other compounds from series showed not significant activity (MIC = 25 or >25 µg/mL).

**Figure 56:**
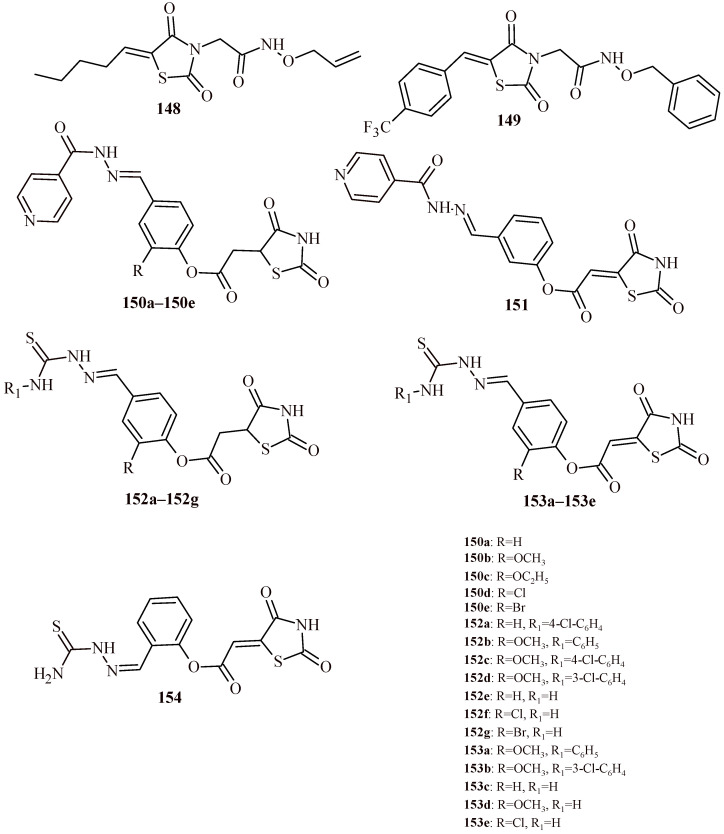
Thiazolidine-2,4-dione derivatives with antitubercular activity.

Šlachtova et al. conducted design and synthesis of a series of thiazolidine-2,4-dione-hydroxamate hybrids [149]. This series was evaluated for their inhibitory activity against recombinant *M. tuberculosis* Zmp1. Nine of them inhibited enzymatic reaction at 78.1–99.8%, that was more effective than phosphoramidon (78% inhibition)—metalloprotease inhibitor. The weak extracellular antimycobacterial activity against *M. tuberculosis* H37Ra was shown by tested compounds. The MIC for most active Compound **148** was 61.8 μM. However, macrophage infection assay revealed, that most of compounds inhibited the intracellular growth of *M. tuberculosis* at a concentration of 10 μM (Compound **149**), whereas it lacks a significant extracellular activity (Figure 56).

Two series of thiazolidine-2,4-dione based derivatives (**150a**–**150e**, **151** and **152a**–**152g**, **153a**–**153e**, **154**) were obtained (Figure 56) [150,151]. These compounds were tested for their antimycobacterial activity against *M. tuberculosis* H37Ra strain. The isoniazid-thiazolidine-2,4-dione hybrids (**150a**–**150e**, **151**) showed promising antimycobacterial activity with MIC = 1 µg/mL. The Compounds **152a**–**152d**, **153a**, **153b** and **154** from series of TZD-thiosemicarbazone derivatives exhibited good antimycobacterial activity at MIC = 2–4 µg/mL. All these derivatives, except **154**, contained phenyl or substituted phenyl group in thiosemicarbazone fragment. The unsubstituted compounds in thiosemicarbazone moiety (**152e**–**152g** and **153c**–**153e**) showed most significant antimycobacterial effects. Their MIC values were in the range of 0.031–0.125 µg/mL, that were better than reference drugs—isoniazid, streptomycin, ethambutol and thiacetazone. All derivatives with highest antimycobacterial effects (**152e**–**152g** and **153c**–**153e**) revealed IC_50_ for Vero cell line higher than rifampicin and selectivity index (SI) significantly higher than 10 it confirms their great potential in biomedical applications.

Worth noticing good antimycobacterial activity of Compounds **113a**–**113d** (Figure 49) that inhibited mycobacterial growth of *M. tuberculosis* H37Rv strain at MIC = 1.56 µg/mL [120].

The very promise level of antimycobacterial activity against *M. tuberculosis* H37Rv showed compounds **42c** (MIC = 1.5 µg/mL) and **43g** as well as **43h** (MIC = 0.8 µg/mL) (Figure 18) [25,26].

### 2.9. Antiviral Activity

**Figure 57:**
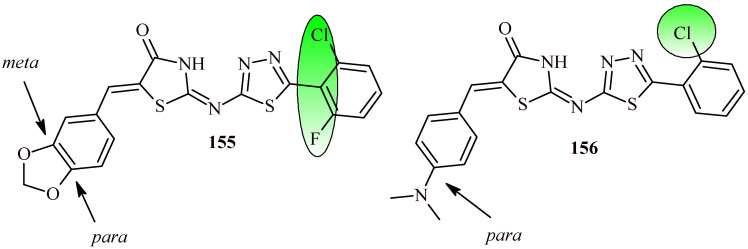
The structures of thiazolidin-4-one-1,3,4-thiadiazoles with activity against HCV.

Al-Behery et al. conducted evaluation series of thiazolidin-4-one-1,3,4-thiadiazole hybrids against hepatitis C virus genotype 4a [152]. The results confirms that presence of 2-chloro-6-fluorophenyl and 2-chlorophenyl substituents (green color in Figure 57) have better inhibition effect of HCV NS5B GT4a than presence 3-fluoro, 4-fluoro and 4-chloro substituents in benzene ring of 1,3,4-thiadiazole heterocycle. Whereas *meta* and *para* substitution in 5-benzylidene moiety of thiazolidin-4-one ring were more favorable for antivirus activity than *ortho* substitution. Among the series of thiazolidin-4-one-1,3,4-thiadiazole hybrids, Compounds **155** and **156** revealed most potent activity with IC_50_ values of 0.338 ± 0.01 and 0.342 ± 0.01 µM, respectively.

Chitre et al. published the study, which aimed to designing of thiazolidin-4-one pharmacophore using QSAR studies for anti-HIV activity [153]. The 3D QSAR studies revealed, that presence of electron-withdrawing substituent such as halogenaryl in Position 2 of thiazolidin-4-one ring led to increased anti-HIV activity. The authors suggested that similarly substitution of halogenaryl moiety and sterically less bulky groups on the *N*-3 position of thiazolidinone ring would be preferred for increased anti-HIV activity among 2,3-diarylthiazolidin-4-ones [153].

### 2.10. Cerebrovascular Protection Activity and Properties against Neurological Disorders

**Figure 58:**
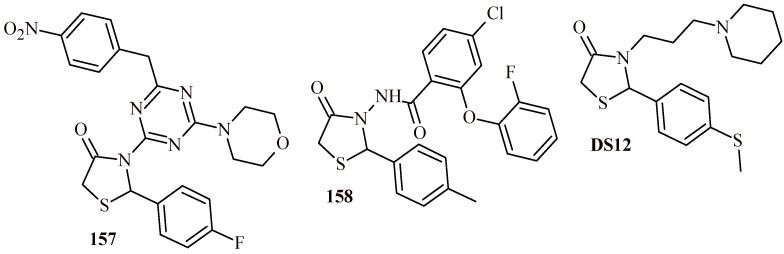
The 2-aryl thiazolidin-4-ones with cerebrovascular protection properties and used in neurological disorders.

Lu et al. carried out study aimed to evaluation of thiazolidin-4-one-1,3,5-triazine hybrids as protective agents towards cerebral ischemia-reperfusion injury. The derivatives showed great in vitro inhibition of NF-kB activation in RAW264.7 cells. The most effective inhibitor of NF-kB (Compound **157**, IC_50_ = 0.90 ± 0.12 µM) was tested in the in vivo experiment (Figure 58). The cerebral ischemia-reperfusion injury induced by middle cerebral artery occlusion. The results demonstrated that Compound **157** showed neuro-protective effect in mice through attenuation of inflammation, oxidative stress and apoptosi. This compound (**157**) inhibited activation of NF-kB pathway [154].

Compound **158** as novel benzodiazepine agonist showed appropriate sedative-hypnotic activity, potent anticonvulsant activity, reduced memory impairment and no muscle relaxant influence in vivo assays. The ED_50_ values were 12.97 mg/kg (maximal electroshock seizures), 15.94 mg/kg (pentabarbital-induced sleeping test) and 21.07 kg/mg (open-field test) [155].

In the study conducted by Silva et al. the antiamnestic effect of DS12 (Figure 58) in a scopolamine-induced memory deficit model in rats was investigated. Compound DS12 at both doses (5 and 10 mg/kg) showed prevention memory loss induced by scopolamine. In the same doses DS12 prevented changes in acetylcholine esterase (AChE) and Na^+^/K^+^-ATPase activity. The DS12 also prevented the increase in AChE activity in lymphoctes after pretreatment at both doses (5 and 10 mg/kg). The protection effect of DS12 on the cerebral cortex and hippocampus from oxidative stress caused by scopolamine confirmed at doses 5 and 10 mg/kg. Therefore, DS12 appeared as a multitarget compound with antioxidative anti-inflammatory and acetylcholine esterase activities [156].

### 2.11. Acetylcholine Esterase, Carbonic Anhydrase and Urease Inhibitors 

**Figure 59:**
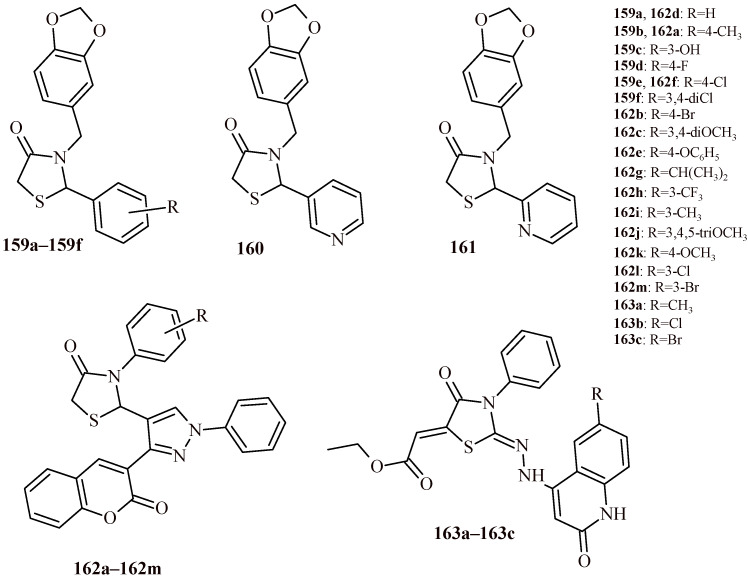
The structures of thiazolidine-4-one as effective AChE, hCA and urease inhibitors.

The piperonal-thiazolidin-4-one hybrids (**159a**–**159f**, **160**, **161** and 4-nitro analogue of **159** (Figure 39)) showed excellent inhibitory activity against AChE at nanomolar concentration in the range of 0.84–2.81 nM. The most active was Compound **159d** (4-fluoro derivative) with IC_50_ = 0.84 nM (Figure 59). Whereas these derivatives inhibited human carbonic anhydrase (hCA) Isoforms I and II at submicro molar concentration (IC_50_ = 91–334.3 nM) [92].

As reported Thacker et al., thiazolidine-4-one coumarin hybrids (**162a**–**162m**) showed inhibitory activity against hCA with good selectivity to hCA IX towards hCA XII. The most effective was Compound **162i** that inhibited hCA IX with K_i_ = 61.5 nM. The K_i_ for hCA XII was 586.6 nM [157].

The Compounds **163a**–**163c** exhibited urease inhibitory activity with IC_50_ values 0.92, 0.74 and 0.46 µM, respectively [158].

## 3. Conclusions

This article provides the summarizing overview of the recent information about antioxidant, anticancer, anti-inflammatory, analgesic, anticonvulsant, antidiabetic, antiparasitic, antimicrobial antitubercular and antiviral activities published in 2020 and 2021. The thiazolidin-4-one system is highly effective in the above-mentioned kinds of biological activity. Additionally, some of them showed dual-target or multitarget activity. These properties are desirable in the treatment of complex diseases such as diabetes, cardiovascular diseases, neurodegenerative syndromes or cancer. Therefore, this review may be useful for further development of the thiazolidin-4-one derivatives group as potential bioactive agents.

## Figures and Tables

**Table 1:** Anticancer activity of the synthesized compounds (**21a**–**21e**) against MCF-7 and A549 cell lines.

Compound	IC_50_, µM	
	MCF-7	A549
**21a**	4.05 ± 0.09	0.72 ± 0.03
**21b**	3.76 ± 0.08	5.31 ± 0.2
**21c**	2.30 ± 0.05	21.3 ± 0.9
**21d**	7.49 ± 0.16	3.22 ± 0.1
**21e**	1.003 ± 0.02	44.7 ± 1.8
erlotinib	11.18 ± 0.24	10.0 ± 0.4
gefitinib	3.57 ± 0.09	3.53 ± 0.1

**Table 2:** The anticancer activity of quinolinone-thiazolidin-4-one hybrids **23** and **24a**–**24d**.

Compound	IC_50_, µM		
	MDA-MB-231	MCF-7	HEK-293
**23**	17.68	39.38	> 1000
**24a**	18.75	24.72	> 1000
**24b**	8.16	33.86	846.93
**24c**	44.24	18.03	409.72
**24d**	22.20	36.98	682.12
cisplatin	1.66	4.99	NT

NT—not tested.

**Table 3:** Effects of Compounds **28a** and **28b** on the expression of CDK1, p53, caspase-3 and caspase-9.

Compound	IC_50_, µM ± S.E.			
	CDK1	p53	Caspase-3	Caspase-9
**28a**	1.13 ± 0.08	1.07 ± 0.33	1.19 ± 0.32	0.93 ± 0.27
**28b**	0.38 ± 0.04	3.04 ± 0.22	5.60 ± 0.49	3.65 ± 0.47
doxorubicin	0.42 ± 0.07	2.36 ± 0.25	3.41 ± 0.25	4.17 ± 0.60

**Table 4:** Cytotoxic activity of Compounds **34**–**36** against HT-1080, A549, MCF-7 and MDA-MB-231 cell lines.

Compound	IC_50_, µM			
	HT-1080	A549	MCF-7	MDA-MB-231
**34**	16.93	20.13	15.56	22.02
**35a**	17.04	19.42	25.30	27.65
**35b**	17.15	15.39	27.85	21.04
**35c**	16.60	15.04	19.89	27.87
**36**	18.75	23.54	19.69	28.41
doxorubicin	4.45	5.28	6.32	5.16

**Table 5:** The cytotoxic activity of bis-thiazolidin-4-ones against HepG2, MCF-7 and Caco-2 cell lines.

Compound	IC_50_, µM		
	HepG2	MCF-7	Caco-2
**40a**	36.07	22.04	45.91
**40b**	34.94	28.49	53.97
**40d**	61.67	33.34	47.19
**41c**	43.04	50.69	67.59
doxorubicin	0.318	1.28	0.332

## Abbreviations

15-LOX—15-lipooxygenase; 5-LOX—5-lipooxygenase; A2780—ovarian carcinoma cell line; A549—lung cancer cell line; AChE—acetylcholine esterase; ACHN—renal cancer cell line; AGS—gastric cancer cells; AKT—serine/threonine protein kinases; AR—aldose reductase; B16F10—mouse melanoma cells; Ba/F_3_—leukemia cell line; BALB/c—laboratory-bred strain of the mouse; BK S-db/db—diabetic mouse model; Caco-2—colorectal adenocarcinoma cells; CAKI-1—renal cancer cell line; CC_50_—cytotoxic concentration; CCRF-CEM—human leukemic lymphoblasts; CDK1—cyclin-dependent kinase 1; CEAC—vitamin C equivalent antioxidant capacity; c-Met—tyrosine-protein kinase Met; CNS—central nervous system; COX—cyclooxygenase; COX-1—cyclooxygenase-1; COX-2—cyclooxygenase-2; Dami—leukemia cell line; DLD-1—human colon cancer cells; DPPH—2,2-diphenyl-1-picrylhydrazyl; DU145—prostate cancer cell line; EC_50_—half maximal effective concentration; ED_50_—median effective dose; EGFR—epidermal growth factor receptor; hCA—human carbonic anhydrase; HCT116—human colon cancer cell line; HCV NS5B GT4a—hepatitis C virus NS5B polymerase of GT4a; HEK-293—human embryonic kidney cells; HDAC—histone deacetylase; HeLa—cervical cancer cells; HepG2—human liver cancer cell line; Het—heteryl; HGF-1—human gingival fibroblasts; HIF-1α—hypoxia-induced factor-1α; HIV—human immunodeficiency virus; HL-60—leukemia cell line; *Hs*MetAP1—human methionine aminopeptidase 1; HT-1080—fibrosarcoma cell line; HT-29—human colon cancer cell line; IC_50_—half maximal inhibitory concentration; IC_90_—the concentration causing 90% growth inhibition; Jurkat—leukemia cell line; K-562—leukemia cell line; KBM7-SLF N233—leukemia cell line; KBM7_WT—leukemia cell line; K_d_—dissociation constant; K_i_—inhibition constant; LD_50_—median lethal dose; LDL-C—low density lipoprotein-cholesterol; *Ld*MetAP1—*Leishmania donovani* methionine aminopeptidase 1; *Lm*PTR1—*Leishmania major* pteridine reductase 1; MABA—microplate alamar blue assay; MAO—monoamine oxidase; MBC—minimum bactericidal concentration; MCF-7—human breast cancer cell line; MDA-MB-231—human breast cancer cells; MIC—minimum inhibitory concentration; MOLT-4—leukemia cell line; mTOR—mammalian target of rapamycin; MTT—colorimetric assay for assessing cell metabolic activity, MTT—tetrazolium dye; NCI DTP—National Cancer Institute Development Therapeutic Program; NF-kB—nuclear factor kappa-light-chain-enhancer of activated B cells; NO—nitric oxide; p53—tumor protein; PC3—prostate cancer cell line; PDB—Protein Data Bank; PDGFRα—platelet-derived growth factor receptor α; PDGFRβ—platelet-derived growth factor receptor; PIM-1—serine/threonine-protein kinase; PIM-2—serine/threonine-protein kinase; PPARγ—peroxisome proliferator activated receptor γ; PTP1B—protein tyrosine phosphatase 1B; PTR1—pteridine reductase 1; QSAR—quantitative structure-activity relationship; RAW264.7—macrophage cells; RNA—ribonucleic acid; RXF393—renal cancer cell line; SAR—structure-activity relationship; scPTZ test—subcutaneous pentylenetetrazol seizure *test*; SF-295—glioblastoma cell line; SF-539—CNS cancer cell line; SHSY-SY—neuroblastoma cells; SI—selectivity index; SK-MEL-5—melanoma cell line; SK-MEL-28—melanoma cell line; SR—leukemia cell line; SW-620—colon cancer cell line; TBARS—thiobarbituric acid reactive substances; TNFα—tumor necrosis factor α; TYRP-1—tyrosinase related protein 1; TZD—thiazolidine-2,4-dione; UO-31—renal cancer cell line; VEGF—vascular endothelial growth factor; VEGFR-2—vascular endothelial growth factor receptor-2; WI-26 VA4—human diploid lung fibroblasts; Zmp1—zinc-dependent metalloprotease 1.
